# Current status and prospects of computer vision-based attitude and deformation measurement applications in wind tunnels

**DOI:** 10.1038/s41598-025-96000-y

**Published:** 2025-04-07

**Authors:** Xiang Xiangyi, Wang Shuai, Cheng Xu, Zhang Jiang, Meng Fanwu

**Affiliations:** 1 School of Mechanical Engineering, Beijing Institute of Technology, Beijing, 100081 China; 2 China Academy of Aerospace Aerodynamics, Beijing, 100074 China

**Keywords:** Computer vision, Test of wind tunnel, Model attitude, Pose estimation, Deformation measurement, Data fusion, Deep learning, Engineering, Mathematics and computing

## Abstract

Computer vision-based model attitude and deformation measurement is essential in wind tunnel testing, offering benefits such as non-contact operation, automation, visualization, flexible configuration, and high near-field accuracy. However, the specific conditions of wind tunnel environments impose certain limitations on the practical applications of visual measurement techniques. In response to the intelligent demands of wind tunnel tests, this paper summarizes applications for model attitude and deformation measurement in wind tunnels. Over time, visual measurement techniques in wind tunnels have advanced from simple non-contact methods to real-time attitude and deformation assessments. Current trends highlight a shift towards multi-dimensional data fusion and deep learning techniques, which harness the complementary strengths of various technologies. This integration enables the simultaneous measurement of diverse data, significantly improving the efficiency of wind tunnel tests and achieving higher measurement accuracy.

## Introduction

High-speed aerodynamic research primarily employs three methodologies: wind tunnel testing, numerical simulation, and flight testing. Among these, flight testing represents the most authentic representation of real flight conditions, as it occurs in a free-flight state. However, it faces significant challenges, including the inability to directly measure aerodynamic parameters, high costs, lengthy testing cycles, and limited measurement method support. In contrast to numerical simulation, wind tunnel testing offers a more realistic physical environment and accurately captures the objective flow processes, making it the predominant method for high-speed aerodynamic research. Due to the sensitive nature of most wind tunnel tests, particularly those related to military applications, there is a scarcity of publicly available studies utilizing computer vision-based measurements. At the same time, the sensitive nature also underscores the critical importance of developing visual measurement techniques within wind tunnels. Consequently, it is essential to advance research focused on computer vision-based measurement methodologies in wind tunnel environments.

A wind tunnel is a tube-shaped facility that generates and controls airflow to simulate gas flow around an object. It allows researchers to measure aerodynamic forces and observe mechanical phenomena in controlled conditions. Wind tunnel experiments are a common approach for researching and verifying the aerodynamic characteristics of the designed aircraft, which aim to simulate the actual flow conditions, measure the aerodynamic parameters of the scaled-down model of the aircraft, and finally obtain the aerodynamic characteristics of the plane in actual flight based on the similarity criterion^1^. The verification of the actual dynamic characteristics can determine whether the designed aircraft achieves the expected goals and make corrections for optimization. Thus, obtaining the aerodynamic parameters of the aircraft model in the wind tunnel is the foundation for the rational design of the aircraft. Meanwhile, the wind tunnel test data’s accuracy directly determines the aircraft design’s reliability.

As the aircraft design level improves, the aerodynamic characteristics of the aircraft keep enhancing, which poses higher demands on the accuracy of wind tunnel tests and promotes the advancement of wind tunnel experimental data measurement technology. Wind tunnel traditional measurement techniques include gyroscope^2^, strain gauge balance^3^, angle sensor^4^, and fiber grating^5^, of which the first three methods are contact measurement, which is directly in contact with the model, and therefore will inevitably affect the model stiffness and strength, in addition to the pressure of contact process on the model to produce torsion and bending coupling force, so that the measurement process becomes complex. Fiber grating is a non-contact measurement method. However, this approach requires pasting the bare fiber grating on the surface of the model or embedding it in the interior. The fragility of the fiber grating and the large wind load in the wind tunnel can easily cause damage to the fiber grating, resulting in inaccurate measurements. Therefore, it is necessary to encapsulate the fiber grating. However, pasting the encapsulated fiber grating on the model will change the appearance of the model, which will have an impact on the flow field in the wind tunnel and lead to inaccurate measurement results. As a result, it is necessary to develop a non-contact measurement method that does not change its appearance and is suitable for wind tunnel tests.

The advent of computer vision has opened up new avenues for the measurement of data in wind tunnel tests. The fusion of computer vision technology into wind tunnel tests can address the limitations of traditional measurement techniques^6^. Historically, computer vision-based measurement technology has primarily concentrated on assessing flow field characteristics within wind tunnels, resulting in a notable gap in non-contact methods for evaluating model attitude and deformation. However, advancements in computer vision technology have increasingly facilitated the application of visual measurement in wind tunnel environments, enabling precise non-contact evaluations of model attitude and deformation. A typical stereo vision system comprises an industrial high-speed camera (CCD or CMOS), targets, a light source, and a computer for image analysis and processing. Once the vision system is installed and configured, real-time access to digital images is achieved, and image processing is performed automatically by the computer. Typically, multiple images are collected once, with the number of images determined by the camera’s frame rate. The parameters in units of pixels can be converted to units of millimeters by a scale factor determined by the physical size and pixel width of an object at the same depth in the video frame^7^. The core challenge of the application of vision measurement is to establish the geometric transformation relationship between the image plane under the camera coordinate system and the three-dimensional space under the world coordinate system. By utilizing the established geometric transformation relationship, it is possible to derive the shape, size, position, and attitude data of the model through image analysis^8^. Targets-based stereo vision systems for wind tunnel measurements are generally constrained by specific environmental conditions, in addition to the limitations of the wind tunnel equipment itself, such as limited optical access, which confines visualization of the model only by the observation window. Furthermore, there are also some restrictions imposed by the wind tunnel test conditions, including high pressure and rapid airflow rates^9^. However, the ease of configuration, flexibility, and relative simplicity of the structure enable the system to achieve high-accuracy measurements despite these limitations. To further enhance the efficiency and comprehensiveness of non-contact measurements in wind tunnels, various techniques are beginning to converge. By integrating different techniques based on computer vision, it becomes possible to measure both the attitude and deformation of a model while simultaneously assessing the flow field of the wind tunnel. This approach reduces the preparation time required for measurements between different tests, and the data obtained from the various techniques can serve as validation for one another, compensating for each other’s limitations.

This paper focuses on the application of computer vision-based techniques for measuring model attitude and deformation in wind tunnels. It reviews the evolution of early technologies in this domain, summarizes the current applications and their limitations, and analyzes the future development prospects of these technologies. Different from reference 8, it mainly focuses on basic principles and error analysis, which has been published 10 years ago. Following the introduction, “Computer Vision-Based Measurement Technology in Wind Tunnel” Section outlines the brief history and components of computer vision-based measurement systems in wind tunnels, as well as the targets employed in these measurement systems. “A theoretical model of visual measurement” Section presents the theoretical model of visual measurement, encompassing both monocular and multi-ocular vision. “Measurement of attitude and deformation in wind tunnel” Section summarizes the application of attitude and deformation measurement in wind tunnels, which categorizes attitude measurement into three types: non-real-time, real-time, and deep learning-based approaches. “Data fusion-based measurement in wind tunnels” Section discusses the integration of measurement with flow diagnostics, visualization technology, and CAD models. “Challenge” Section addresses challenges, including vibrations induced by wind loads and distortion of the observation window. “Perspectives” Section provides an outlook on future developments. Finally, Sect. 8 concludes with the contributions of this paper.

## Computer vision-based measurement technology in wind tunnel

In addition to measuring the airflow field characteristics in the wind tunnel, it is essential to assess the attitude and deformation of the model, which constitutes a primary objective of the wind tunnel test. By measuring the attitude and deformation of the model under wind load, the aerodynamic characteristics of the aircraft can be analyzed, thereby verifying the reasonableness and safety of the aircraft design and providing a test basis for its optimization.

In wind tunnel tests, visual measurement technology involves capturing images of the model using a camera and transferring these images to a computer. The image processing technology is then employed to automatically analyze the information contained in the images, establishing a correspondence between image coordinates and spatial coordinates to determine the attitude and deformation of the model. Based on the number of cameras used, visual measurement systems can be classified as monocular or multi-ocular. The early development of computer vision-based wind tunnel measurement techniques can be traced back to the 1990s with the introduction of the OPTOTRAK 3D optical motion capture system by the Canadian company NDI, which was utilized for measuring model attitude and deformation in wind tunnels^6,10,11^. This system employs three mutually perpendicular line-array CCD cameras to measure the model’s 3D coordinates, based on the geometrical principle that three non-parallel surfaces in space will intersect at a single point, achieving a maximum accuracy of 0.01 mm^12^. However, the OPTOTRAK system is not widely adopted in wind tunnel tests due to its requirement for embedding point light sources into the model’s surface, which can damage the model’s structural integrity and adversely affect its stiffness and aerodynamic characteristics. Additionally, the cameras in this system must be installed at specific angles and distances, complicating on-site installation and calibration. The OPTOTRAK system is limited to capturing a single data target per measurement, necessitating multiple scans to obtain comprehensive data on the model’s attitude and deformation, which is inefficient and insufficient to meet the demands of wind tunnel testing. Laura^13^ analyzed various wind tunnel measurement systems, including the OPTOTRAK system, and suggested improvements in data processing speed, image quality, and automatic target identification. Currently, a vision system that utilizes targets, also referred to as a videogrammetry method, is widely employed in wind tunnels and has demonstrated excellent measurement results^14^.

### Typical computer vision-based wind tunnel measurement system

**Fig. 1:**
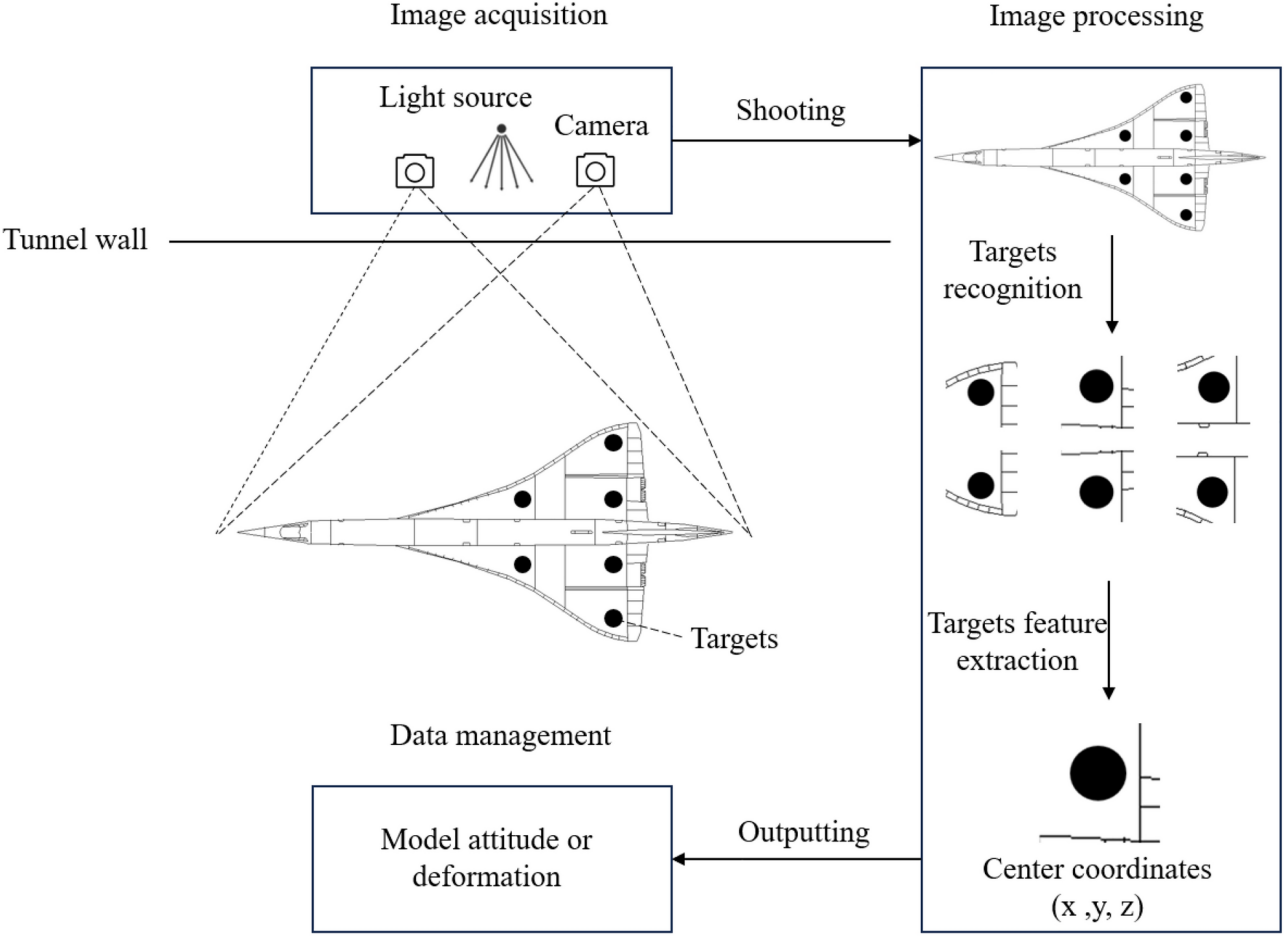
Schematic diagram of wind tunnel test image processing based on computer vision.

A complete wind tunnel measurement system consists of three main components: image acquisition, image processing, and data management^15^. Image acquisition is responsible for capturing images that contain information on model attitude and deformation through an optical system comprising an industrial high-speed camera and a light source. The light source enhances the contrast between critical targets and the model, resulting in a clearer and brighter field of view, which contributes to higher-quality images. This improvement assists the camera in capturing better-quality images, thereby enhancing the accuracy of the measurement system and the efficiency of image processing. However, due to the limited visual optical path of the wind tunnel, it can be challenging or impossible to acquire images of the model from various distances and angles. Consequently, camera calibration and image acquisition may be restricted, sometimes necessitating the use of a single camera. Image processing employs built-in software algorithms to analyze the acquired images and extract information such as the type, shape, and location of targets. Data management involves manually presetting the requirements to display target data derived from a substantial number of image processing results. The wind tunnel measurement system is illustrated in Fig. 1.

### Targets

After completing the selection of cameras and lenses, it is essential to choose appropriate targets based on the model. Targets are critical to achieving high accuracy in the vision system because the essence of analyzing the captured image lies in identifying the targets on the model. Therefore, the choice of target settings significantly impacts both the quality of the images and the reliability of the captured data. By locating the 2D coordinates in the image plane of the target’s center with the projection relationship between the image plane and the 3D space, the 3D coordinates of the target’s center can be determined, which enables the subsequent analysis of the model’s attitude or deformation. Targets commonly used in wind tunnel tests include naturally reflective target^16^, retro-reflective target^17^, white-light dot projection target^18^, fluorescent target, and feature-coded target^19^. There are various shapes of targets, among which circular targets are the most widely used.

Naturally reflective targets come in various types, including white diffuse reflective targets and black diffuse reflective targets, which can be painted or attached to the surface of the model. White and black targets should be used under darker and brighter lighting conditions, respectively, to achieve high contrast and image quality. Retro-reflective targets are less sensitive to light changes and can produce high-contrast images even in low-light conditions. In contrast to naturally reflective targets, the thickness and surface roughness of retro-reflective targets may affect the stiffness and aerodynamic characteristics of the model, which should be taken into account when using it. The white-light dot projection target is a type of contactless marking point that does not impact the model itself. Its projection target falls on the model’s surface, representing the intersection of a specific projector ray with the model’s surface. By calculating the center of each projection target, the three-dimensional coordinates of a series of points can be obtained^20^. Unlike attached targets, projected targets do not move with the model. For attached targets, during the wind tunnel test, the camera captures different spatial coordinates of the same target over time, with each target representing the spatial position of a specific structural point on the model at various moments. In contrast, projected targets allow the camera to capture a series of spatial coordinates for the model as a whole, where the coordinates of the same projected target at different times do not directly correlate with the model’s motion. Instead, the same projection target at different moments is simply located along a straight line, either close to or far from the projector. To determine the model’s attitude or deformation, it is necessary to find the corresponding projection points of the same structural point on the model at different times. The most commonly used coded targets consist of circles and concentric rings, which add coded information to non-coded targets. These targets are characterized by simple features that are easy to recognize, allowing for the matching of corresponding homonymous points by identifying the unique coded information they contain. Additionally, the number of reciprocal targets is sufficient to meet the demands of wind tunnel testing and to expedite the identification and localization process in the visual measurement system, enabling real-time performance.

## A theoretical model of visual measurement

The essence of the computer vision-based measurement system is to use the two-dimensional information from the camera images to obtain the three-dimensional information of the object being measured. The camera imaging process can be described by the small-hole imaging model, which represents the correspondence between the two-dimensional image plane and the three-dimensional spatial coordinates^21^.

**Fig. 2:**
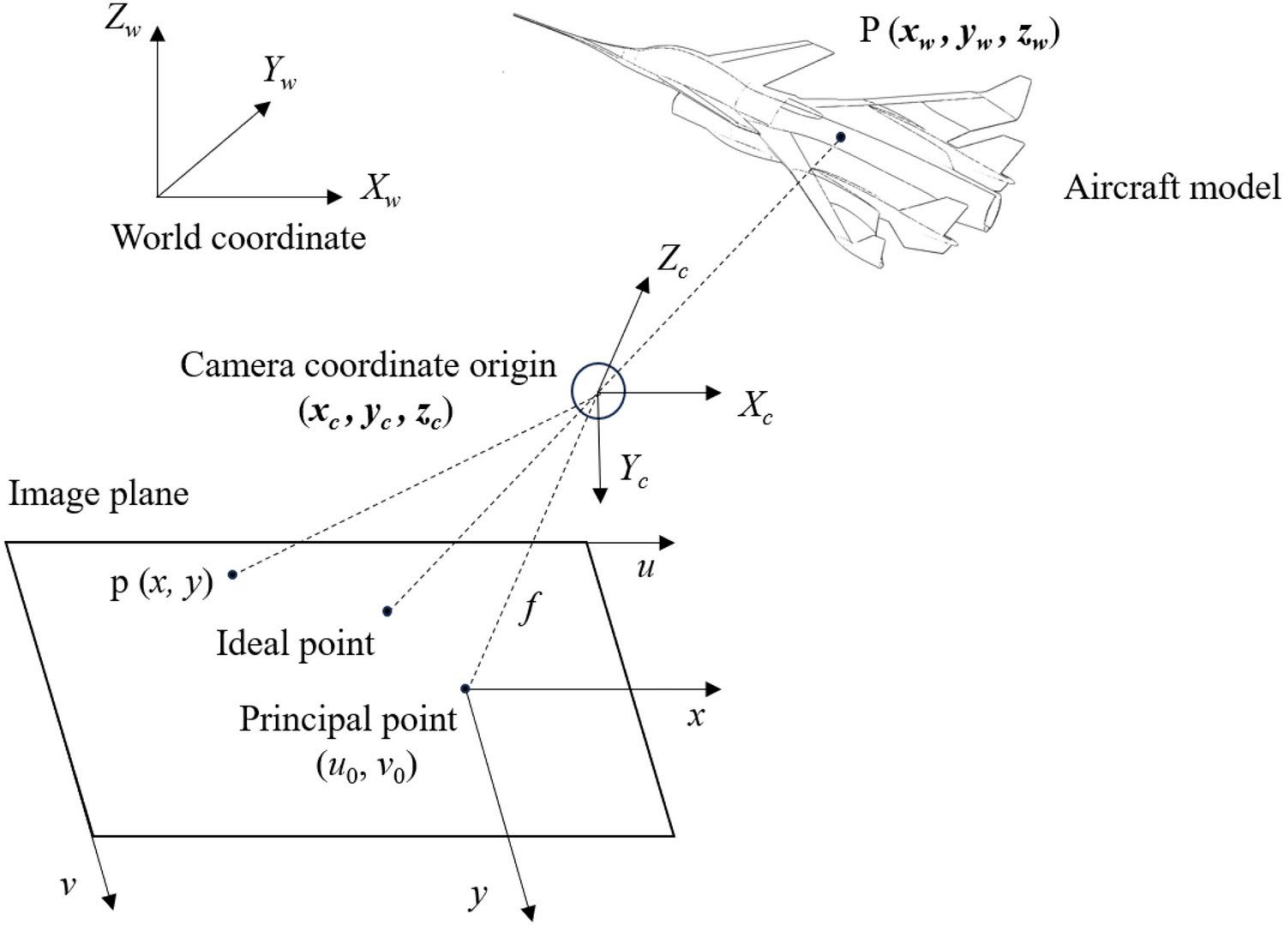
Visual measurement model for test of wind tunnel.

As shown in Fig. 2. P(*x*_*w*_, *y*_*w*_, *z*_*w*_) is a point on the model in the world coordinate system. According to the geometrical projection relationship, the projection point of point P on the image plane is p(*x*, *y*). Due to camera lens imaging aberrations, such as radial and eccentricity aberrations, the projection point of P on the image plane will shift relative to the ideal projection point. The position and orientation of the camera coordinate system concerning the world coordinate system are called external parameters. The origin of the camera coordinate system is the center of focus, whose coordinates in the world coordinate system are (*x*_*c*_, *y*_*c*_, *z*_*c*_). The center of focus essentially treats the camera as a point, establishing a geometric relationship with it as the center of projection. The projection point of the center of focus on the image plane is the principal point (*u*_0_, *v*_0_), and the image coordinate system and the pixel coordinate system do not coincide due to the offset of the principal point. The vertical distance from the camera’s focusing center to the image plane is the focal length *f*. Internal parameters can be determined from the geometric projection relationship between the focusing center and the image plane^22^. The purpose of camera calibration is to determine the internal and external parameters from multiple images with different positions and orientations, as well as to address the imaging aberrations of the lens, facilitating the correction of the captured photographs.

### Single camera measurements

**Fig. 3:**
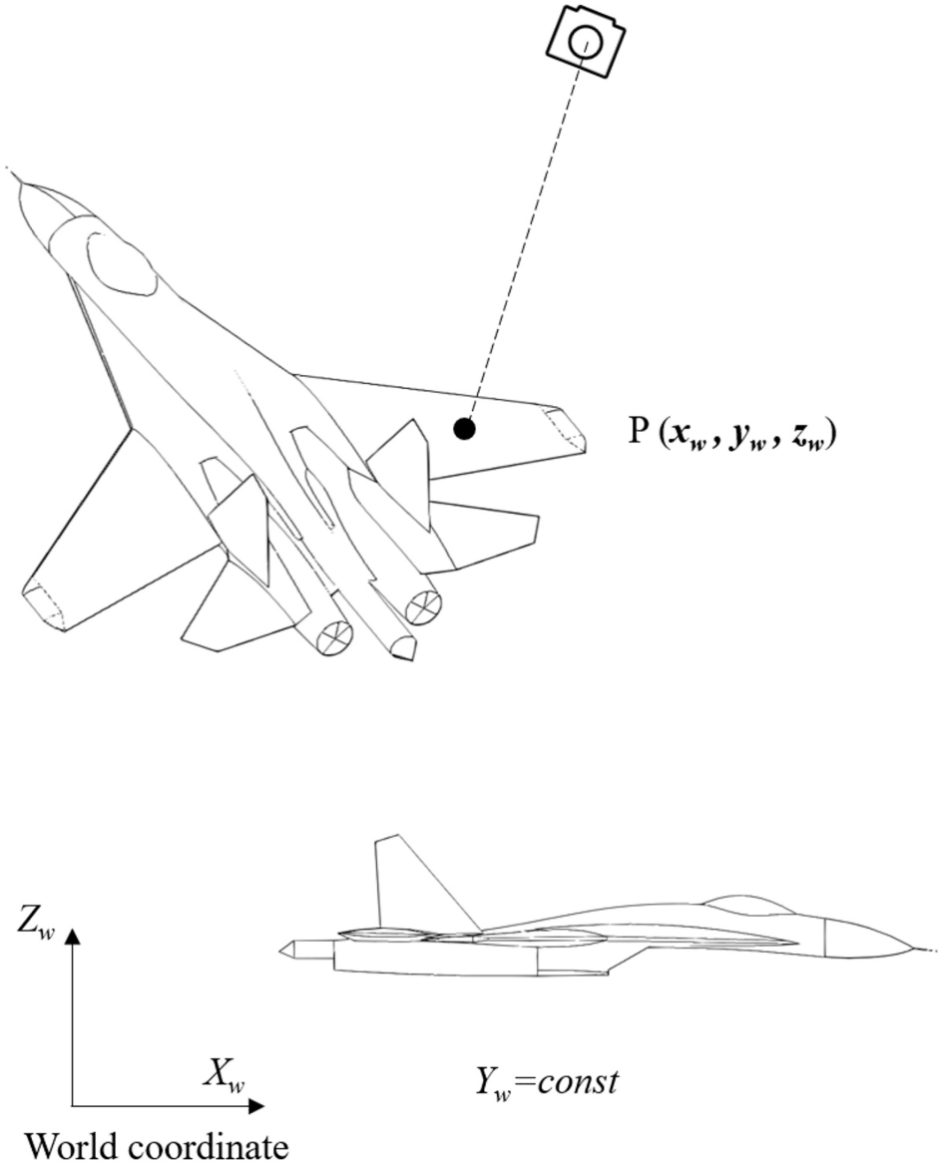
Monocular visual measurement model for wind tunnel test.

In a wind tunnel testing environment utilizing a single camera, it is an inherent challenge to determine the three-dimensional coordinates of targets on the model based solely on a sequence of captured images. This limitation arises because the camera provides only two equations corresponding to the (*u*, *v*) coordinates at each moment in time, making it impossible to uniquely resolve the three unknowns represented by (*x*_*w*_, *y*_*w*_, *z*_*w*_). To address this issue, constraints must be introduced to the model to reduce the number of unknowns, thereby enabling the solution of the remaining two coordinates. In practice, one common approach is to fix the *y*_*w*_ coordinates of the model as constants. This constraint simplifies the equations, allowing for the determination of the other two coordinates, *x*_*w*_ and *z*_*w*_. This methodology is illustrated in Fig. 3 below.

In the figure, the symmetry plane of the aircraft model is parallel to the XOZ plane of the world coordinate system, and the *y*_*w*_ coordinate remains constant over time. Consequently, the model undergoes only a pitching motion under wind load. The advantages of employing a single-camera measurement system include its suitability for measuring the attitude of a model exhibiting a singular pitching motion and the simplicity of its configuration. However, to achieve highly accurate results, it is essential to ensure that the angle between the camera’s optical axis and the plane where the target is located, with a constant value of *y*_*w*_, exceeds 20°^23^. Notably, when this angle is 0°, it becomes impossible to uniquely determine the three-dimensional coordinates of the target.

### Multi-ocular camera measurement

**Fig. 4:**
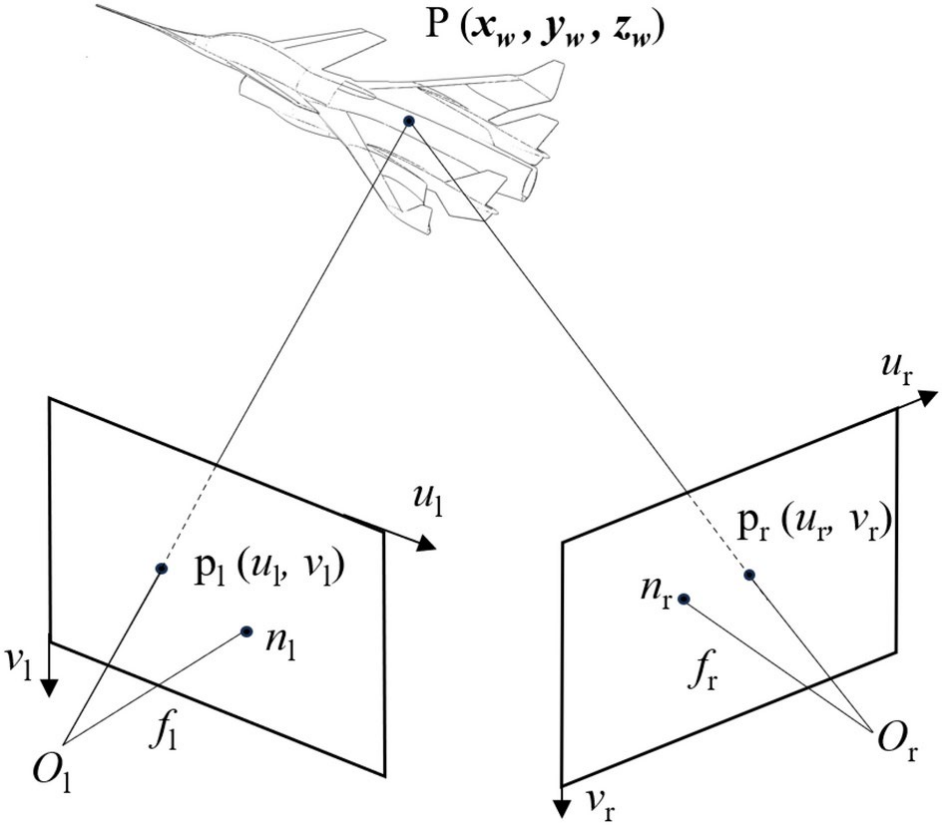
Stereo visual measurement system for wind tunnel test.

Unlike monocular vision, multi-ocular vision refers to a measurement system that utilizes two or more cameras. Taking binocular vision as an example, the three-dimensional coordinates of the unknown target in the image captured at each moment can be derived from equations based on the projection relationship without the need for additional constraints. The principle of which is illustrated in Fig. 4 below.

Where *f* notes the focal length and *n* represents the principal point of the left and right cameras, respectively. Additionally, let p be the imaging point of the three-dimensional coordinates on the model within the two-dimensional image coordinate system. Since binocular vision can determine the three-dimensional coordinates of targets based on known points in the image, it is capable of measuring a variety of motion attitudes and deformations of the model in the wind tunnel test with greater accuracy.

### Camera calibration

During wind tunnel tests, the attitude and shape of the model undergo dynamic changes in response to the wind load. Accurately determining the state of the model hinges on establishing the geometrical relationship between the three-dimensional points in the world coordinate system and the two-dimensional points in the image coordinate system^24^. The objective of camera calibration is to ascertain the internal and external parameters of the vision measurement system and to establish this correspondence effectively.

**Fig. 5:**
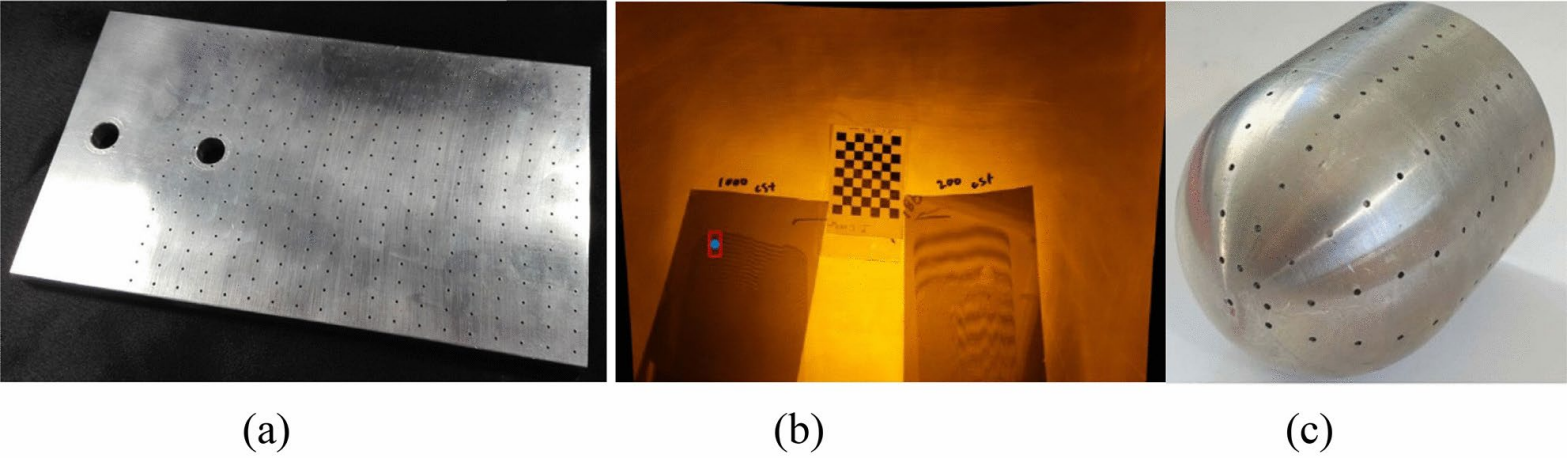
Calibration target used in the wind tunnel: (**a**) board with control holes^28^ (**b**) checkerboard^29^ (**c**) cover with control holes^30^.

Traditional linear algorithms often separate the internal and external parameters of the camera by directly solving the associated equations without iteration, but these methods generally yield lower accuracy^25^. Hallert was the first to implement the least squares method for processing camera calibration parameters. Subsequently, Beyer extracted 3D information and camera parameters from planar images using least squares estimation grounded in standard photogrammetry. Tsai^26^ proposed a two-stage approach based on the Direct Linear Transformation (DLT) algorithm, which first employs linear constraints to resolve external parameters before applying iterative optimization to obtain internal parameters. Zhang^27^ advanced this methodology by utilizing a calibration plate to derive initial estimates for the camera’s internal and external parameters through a single response matrix. He then introduced distortion parameters, refined them using least squares iteration, and ultimately optimized all parameters using maximum likelihood estimation (MLE), which is the most widely adopted calibration technique to date. In contemporary practices, nonlinear iterative optimization methods typically involve transforming the equations into a linear form to procure accurate initial values, followed by iterative optimization processes to refine the results^28–30^, what they used for camera calibration in wind tunnels are shown in Fig. 5.

In wind tunnel testing, a calibration target is placed around the model for camera calibration, however, the camera can only capture images through the observation window on the wall of the wind tunnel. Due to the limited dimensions of this window, aligning the size of the calibration target with the camera’s field of view presents a challenge. Furthermore, once the camera has been calibrated, the image resolution becomes fixed, if the image of the calibration target appears smaller, its effective resolution decreases correspondingly, impacting the accuracy of the parameters derived during calibration.

**Fig. 6:**
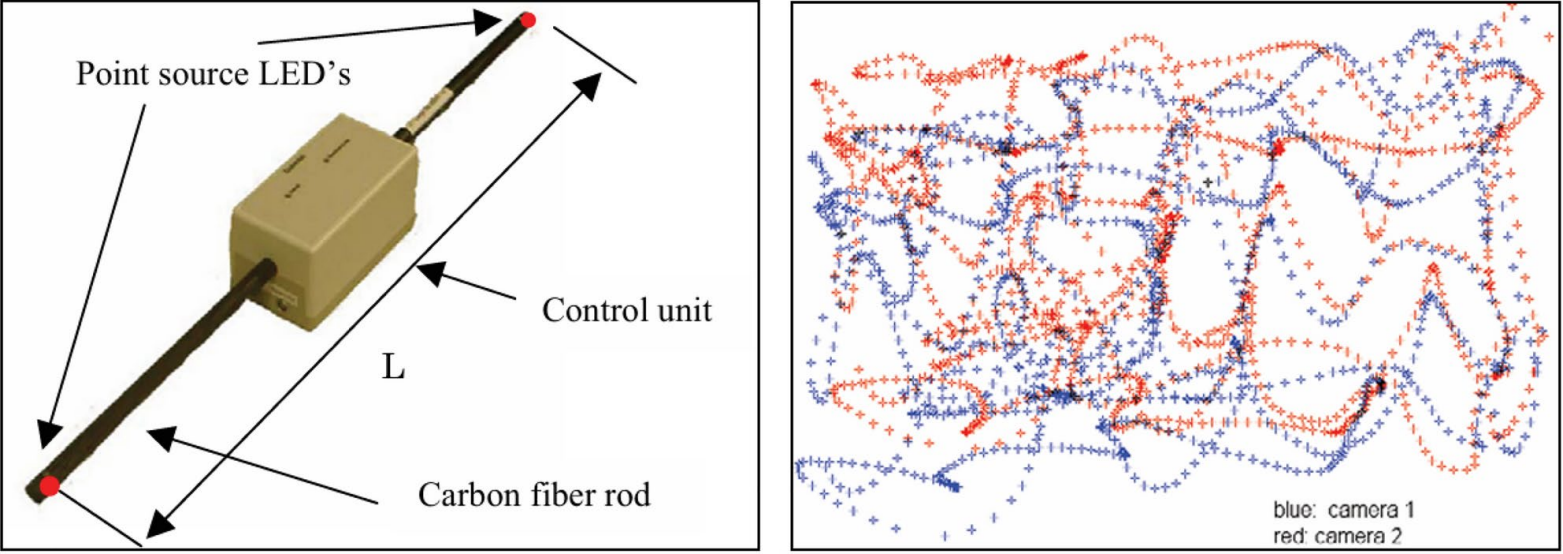
Mobile calibration rod featuring two-point source LEDs mounted and overlay of 600 image pairs produced by the calibration rod^31^.

To address this issue, the camera calibration process can be divided into two distinct steps, effectively performing two separate calibrations. First, the calibration target is placed outside the wind tunnel, ensuring that the camera’s field of view accommodates the entire size of the calibration target. This setup allows the image of the calibration target to fill the camera’s field of view, enabling a more precise determination of the camera’s internal parameters. In the second step, the calibration target is positioned around the model to acquire the external parameters. The internal parameters obtained from the first step remain constant, provided that the camera and lens settings do not change. This approach effectively enhances the accuracy of the camera calibration parameters. Machacek^31^ proposes a two-step method for camera calibration in wind tunnels, utilizing a planar calibration target marked with a point structure and precision-drilled holes of 0.01 mm depth as calibration markers. The first step involves translating the plate to a specified distance to generate a set of three-dimensional calibration points, enabling the determination of the internal parameters. In the second step, a self-calibration algorithm is employed to ascertain the external parameters. This process utilizes a two-point marker comprised of a low-distortion carbon fiber rod and two point-source LEDs that move randomly within the measurement area, as shown in Fig. 6. Each image captured corresponds to the position of the calibration target, from which the external parameters can be derived. Compared to typical wind tunnel calibration methods, this approach achieves a camera orientation accuracy of 0.002°, a positional accuracy of 2.5 mm, a standard deviation of 0.7 mm for length estimation, and a velocity accuracy of up to 0.00006 m/s over a segment length of 10 cm.

## Measurement of attitude and deformation in wind tunnel

### Attitude estimation

The attitude of a model refers to the process of its spatial motion changes, typically recorded during wind tunnel tests using target-based videogrammetric measurements^13,32,33^. Videos are made up of a series of images and thus have two domains: the spatial domain corresponding to a 2D field of brightness values in a single image, and the time or temporal domain corresponding to the image as it evolves in time to make a video^34^. The spatial motion of the model can be quantitatively described with precision by multiple targets attached to the rigid segments of its surface. Attitude estimation based on these point features fundamentally involves solving the Perspective-n-Point (PnP) problem.

#### Non-real-time attitude estimation

In terms of speed criteria, wind tunnel test attitude estimation can be categorized into non-real-time methods and real-time methods. Non-real-time methods rely on the analysis of images captured by the camera through offline post-processing techniques. Ruyten^35^ designed a position and attitude measurement method using trinocular vision in a transonic wind tunnel at the Arnold Center for Engineering Development, which utilizes a simplex iterative method to minimize the sum of squared errors. The determination of the model’s position and attitude parameters using targets with known positions relative to the model enables the fusion of measurements from multiple cameras, even when their fields of view don’t overlap. However, experimental results revealed an error of 0.05° between the angles measured by the camera and the theoretical angles. Additionally, factors such as camera vibration were identified as influencing the measurement results. Chen^36^ proposed a visual measurement method utilizing monocular vision for a model mounted on a wire-driven parallel suspension system. This suspension system effectively reduces airflow interference, thereby making the wind tunnel test environment more representative of real flight conditions. The method employs the generalized inverse least squares method through HALCON software to measure the model’s attitude. However, only three landmarks are used for feature extraction in this method, and the experimental results may have accidental errors.

**Fig. 7:**
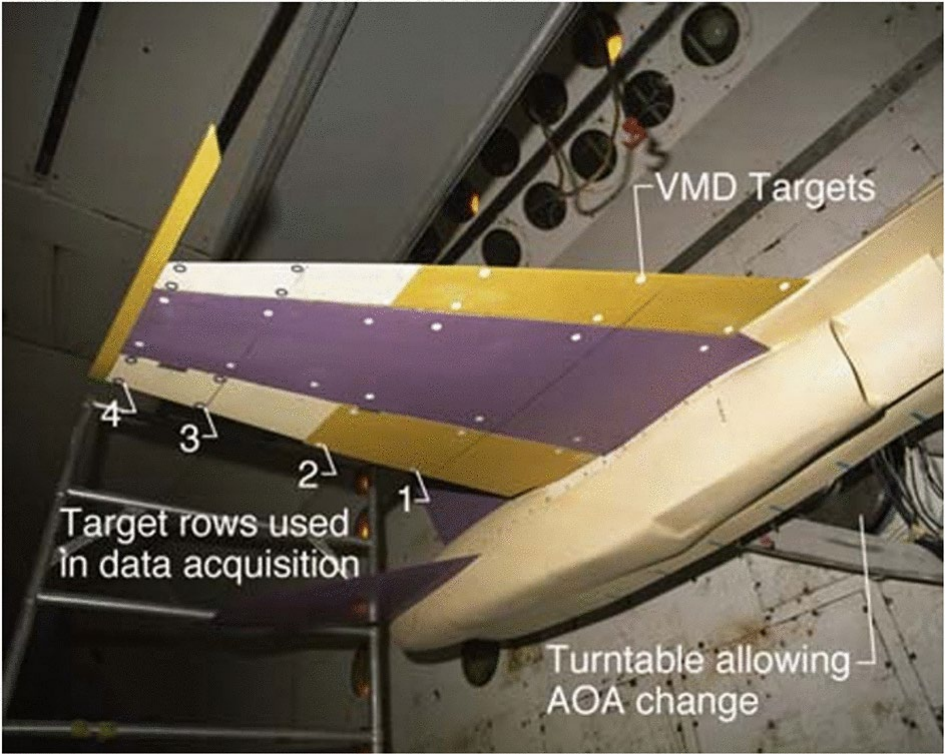
Model of the aircraft and distribution of targets^37^.

Spain^37^ measured pitch angle, displacement, and model deformation at different Mach numbers and dynamic pressures, there were anomalies in some of the data points in the experiment that deviated too much from the theoretical values, by analyzing the experimental results found that a structural failure occurred in the control surfaces, affecting the experimental process and the efficiency of the measurements, which could be avoided under real-time detection. The distribution of targets in the model is shown in Fig. 7. Shewhart’s variable control charts (also known as statistical quality control charts) were used to assess the consistency and uncertainty of the videogrammetric model deformation (VMD) system. This included ${\overline{\text{X}}}$ and R control charts to assess the consistency of the data and to analyze the variability of individual samples. ${\overline{\text{X}}}$ and R present the measurement average and the X_maximum_ minus X_minimum_ for each group. The experiments described in this paper utilize calibration blocks for calibration. While this method achieves high accuracy, the calibration process is complex and the installation poses challenges. Leifer^38^ discusses the measurement of acceleration in wind tunnel tests using videogrammetric measurement. Since acceleration can’t be obtained directly from visual measurements and requires differentiation twice based on displacement, mathematical filtering is applied using Savitzky and Golay algorithms to minimize noise generated during the differentiation process.

#### Real-time attitude estimation

**Fig. 8:**
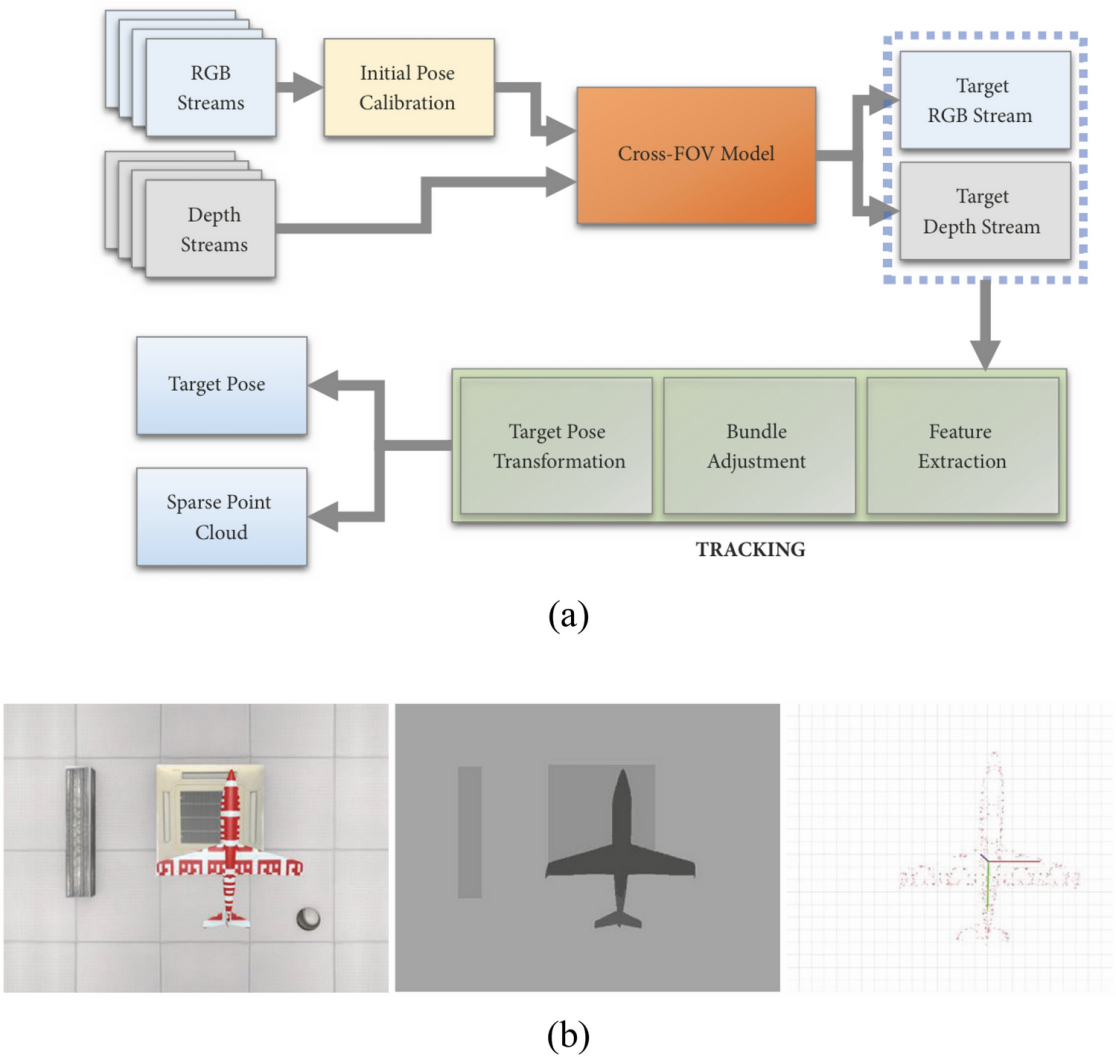
The real-time RGB-D method^40^: (**a**) overview of the method showing the data-flow starting from RGB-D streams (**b**) the synthetic RGB-D input and the estimated point cloud result of the system.

Real-time attitude estimation not only enhances speed but also improves test efficiency by monitoring the validity of the data during the experiment. Thomas^39^ designed a binocular vision-based model attitude measurement system for supersonic wind tunnels, employing the least squares method to determine the model’s position and attitude while investigating the impact of vibration on measurement accuracy, results of the method are presented in Fig. 12a. To address the inconvenience of moving calibration targets and prevent delays in wind tunnel operations, a system utilizing a fixed calibration plate with beveled edges is proposed, eliminating the need to remove the camera for in-situ calibration. The camera is fixed at the top of the wind tunnel, and the vibration of the wind tunnel equipment will change the external parameters of the camera and introduce errors. Liu^40^ introduced a real-time attitude estimation system for low-speed wind tunnels utilizing trinocular RGB-D cameras across the field of view (FOV), as shown in Fig. 8. The system begins by developing a multimodal initialization method to measure the spatial relationship between the camera and the aircraft. It then automatically and accurately acquires images using a cross-FOV model and locates the center of the AprilTag. Next, image features are extracted using a fast binary descriptor called ORB, through a bundle adjustment process to minimize reprojection error. Finally, the model’s sparse point cloud and attitude information are obtained. Results of the method are shown in Fig. 12b. However, when the wing is deformed, there are errors in the process of determining the center point of the model.

**Fig. 9:**
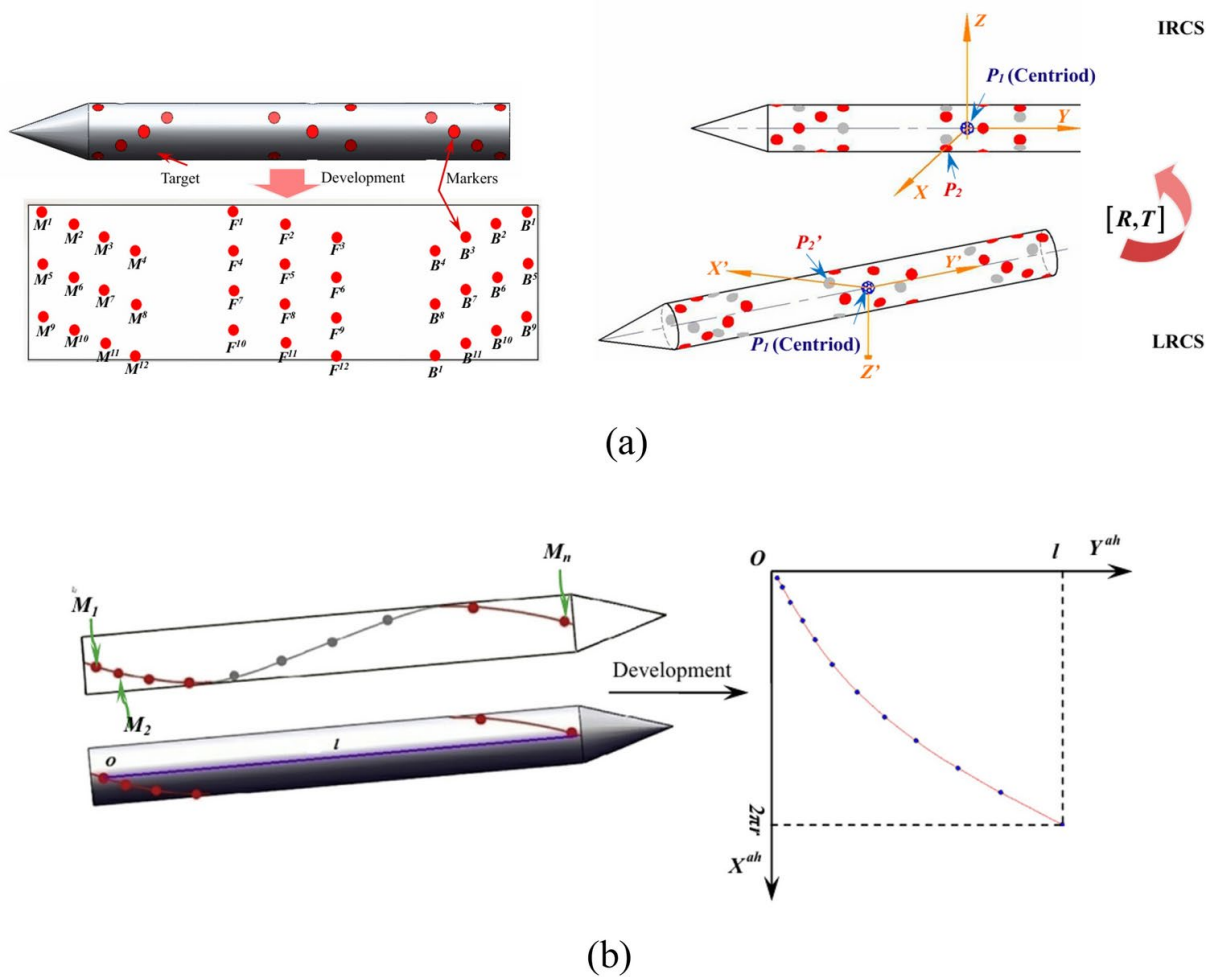
The target layout: (**a**) a spatial encoding layout and Schematic of the principal of absolute pose solution^41^ (**b**) an acceleration–helix layout and the outspread curve of the acceleration–helix layout^42^.

In addition to the linear layout of the targets, a reasonable spatial layout will enhance the accuracy of the measurement. Zhen^41^ proposed a case deletion diagnostic measurement method based on videogrammetric measurement techniques and binocular vision with a spatial coding layout, as shown in Fig. 9a, to achieve real-time, high-accuracy position acquisition. This case deletion diagnosis is employed to eliminate incorrectly matched target points during the template matching process, ensuring that the measurement system accurately captures valid and effective model pose information. Liu^42^ proposed a real-time position measurement method based on binocular vision for dark and transonic rolling wind tunnel environments. Retro-reflective targets are detected using a background subtraction algorithm, ensuring that high-quality images can still be extracted in low-light conditions, and the center of targets is identified by the centroid method. To address the issue of high-speed model scrolling, the targets are arranged in an acceleration-spiral layout to prevent obscuration during the model’s movement, as shown in Fig. 9b. For these two methods of special target layout, the error of roll is higher than pitch and yaw Angle.

Fan^43^ employed the Optitrack system to assess the model’s attitude within a low-speed wind tunnel. Initially, the system’s accuracy was evaluated through ground static experiments, which demonstrated that the static position accuracy was better than 0.07 mm, while the accuracy of attitude angle measurement reached 0.05°. These results align with the requirements for dynamic test accuracy. Furthermore, the dynamic tests confirmed that the system’s accuracy could achieve up to 0.5°, and the model data updates met the minimum requirement of 100 Hz for free-flight testing. This confirms the system’s potential applicability in free-flight tests and indicates improvements in both test efficiency and data quality. It is important to clarify that the Optitrack system discussed herein is distinct from the OPTOTRAK system. The OPTOTRAK system employs three mutually perpendicular linear array CCD cameras for measurement, whereas the Optitrack system is a motion capture system that allows flexible configurations with multiple high-speed cameras based on the testing requirements, in this case, Fan utilized eight cameras, which is a great challenge for wind tunnel equipment layout. Additionally, the Optitrack system utilizes small and thin targets, measuring 0.12 mm in thickness and 6 mm in diameter. These targets are affixed to the model’s surface without compromising its structural integrity or rigidity. The comparision of attitude estimation methods used in test of wind tunnel is listed in Table 1.

**Table 1:** Comparision of attitude estimation methods used in test of wind tunnel.

	Author	Principles of vision	targets	methods	Test environment	accuracy
Non-real-time	Ruyten^35^	Trinocular vision	Fluorescent	Simplex iteration	Transonic	0.005°the root-mean-square (RMS) pitch and yaw
	Chen^36^	Monocular vision	Natural reflection	Generalized inverse least square	Low-sonic	
	Spain^37^	Monocular vision	Natural reflection		Except for the supersonic	
	Leifer^38^	Trinocular vision	Retro-reflective	Seven-point numerical differentiation		X, Y, Z-axis position accuracy is 0.0079 mm, 0.0144 mm, and 0.0186 mm rms respectively
Real-time	Thomas^39^	Binocular vision	Retro-reflective	Least square	Supersonic	Figure 12a shows the maximum angle error is 0.3°, the average error of pitch Angle is 0.0404, and the prediction interval is 95%
	Liu^40^	Trinocular depth vision	AprilTag	ORB and Bundle Adjustment Algorithm	Low-sonic	Figure 12b shows the angle and displacement are consistent with the sensor
	Zhen^41^	Binocular vision	Retro-reflective	Case Deletion Diagnostics and least square	Supersonic	Position accuracy is 0.16mm, pitch, and yaw angle accuracy is less than 0.132° and roll angle accuracy is 0.712°
	Liu^42^	Binocular vision	Retro-reflective	Centroid method with three-dimensional constraints	Supersonic	Rms errors of 0.182 mm, 0.158°, 0.212°, 0.9° for displacement, pitch, yaw and roll angles
	Fan^43^	Multi-ocular vision	Natural reflection	least square	Low-sonic	Static position accuracy is better than 0.07mm, attitude angle measurement accuracy is up to 0.05°, and dynamic measurement accuracy is 0.5°
Deep-learning	Huang^45^	Monocular vision	Natural reflection	CNN + GAN	Low-sonic	Figure 12c shows the acceleration are consistent with the sensor

#### Deep learning-based attitude estimation

**Fig. 10:**
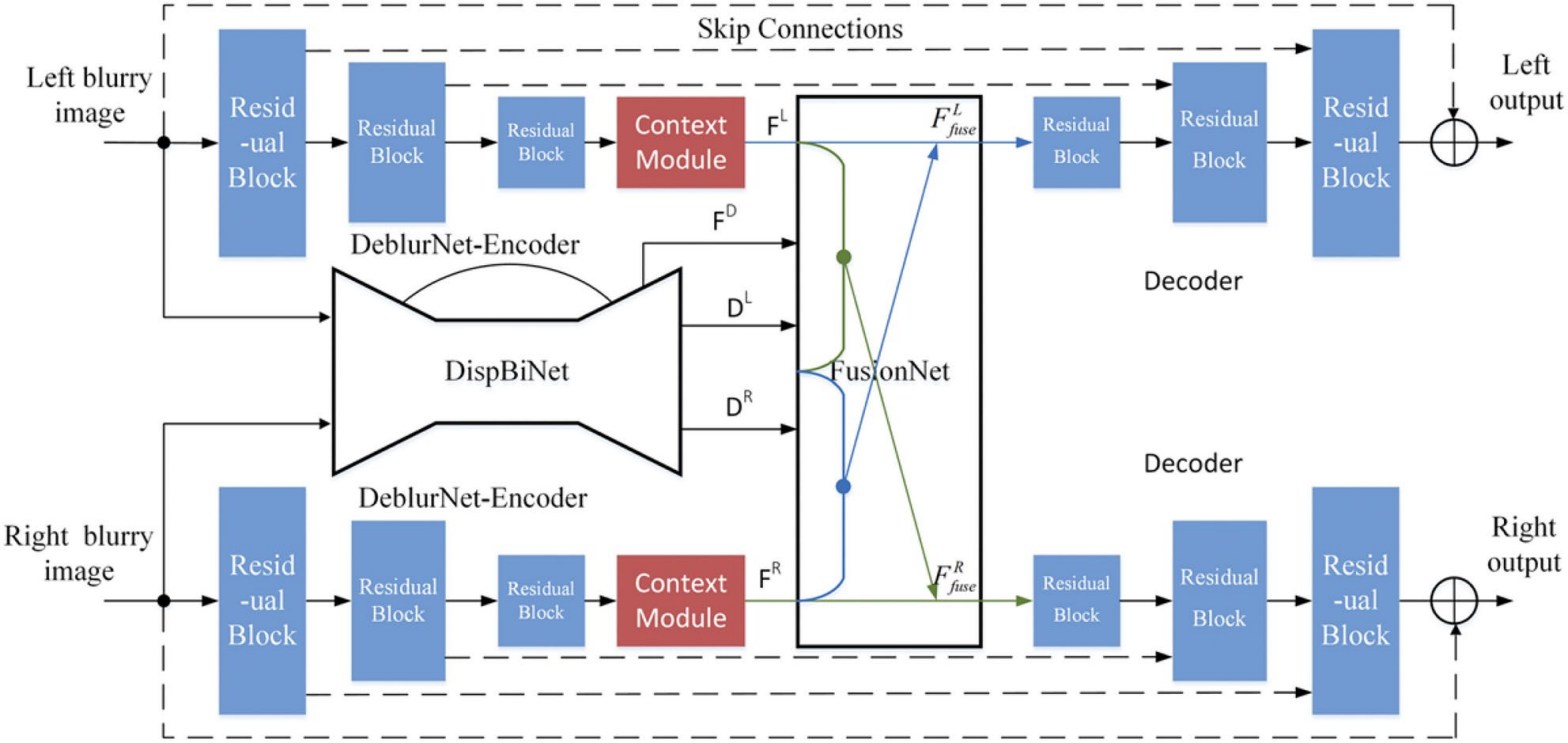
Overall structure of DAVANet^44^.

The high cost and substantial spatial requirements of wind tunnel equipment, coupled with the military confidentiality associated with many wind tunnel tests, have resulted in a limited number of studies focused on wind tunnel testing utilizing computer vision techniques. Furthermore, deep learning approaches generally necessitate a large volume of training data, and the amount of publicly available data from wind tunnel tests is relatively scarce. Currently, there is still a big gap in wind tunnel attitude measurement based on deep learning. The few researchs have been done on deep learning applications in wind tunnels primarily revolves around point features, employing neural networks to predict the 2D–3D correspondence of these features to determine target positional parameters. For instance, Zhang^44^ utilized DAVANet to mitigate image blur, as shown in Fig. 10. However, a notable drawback of those approaches is that the neural network is optimized merely for feature extraction and matching during training, which doesn’t fully leverage its advantages for measurement tasks. Nonetheless, this limitation underscores the significant untapped potential for the application of deep learning within this domain.

**Fig. 11:**
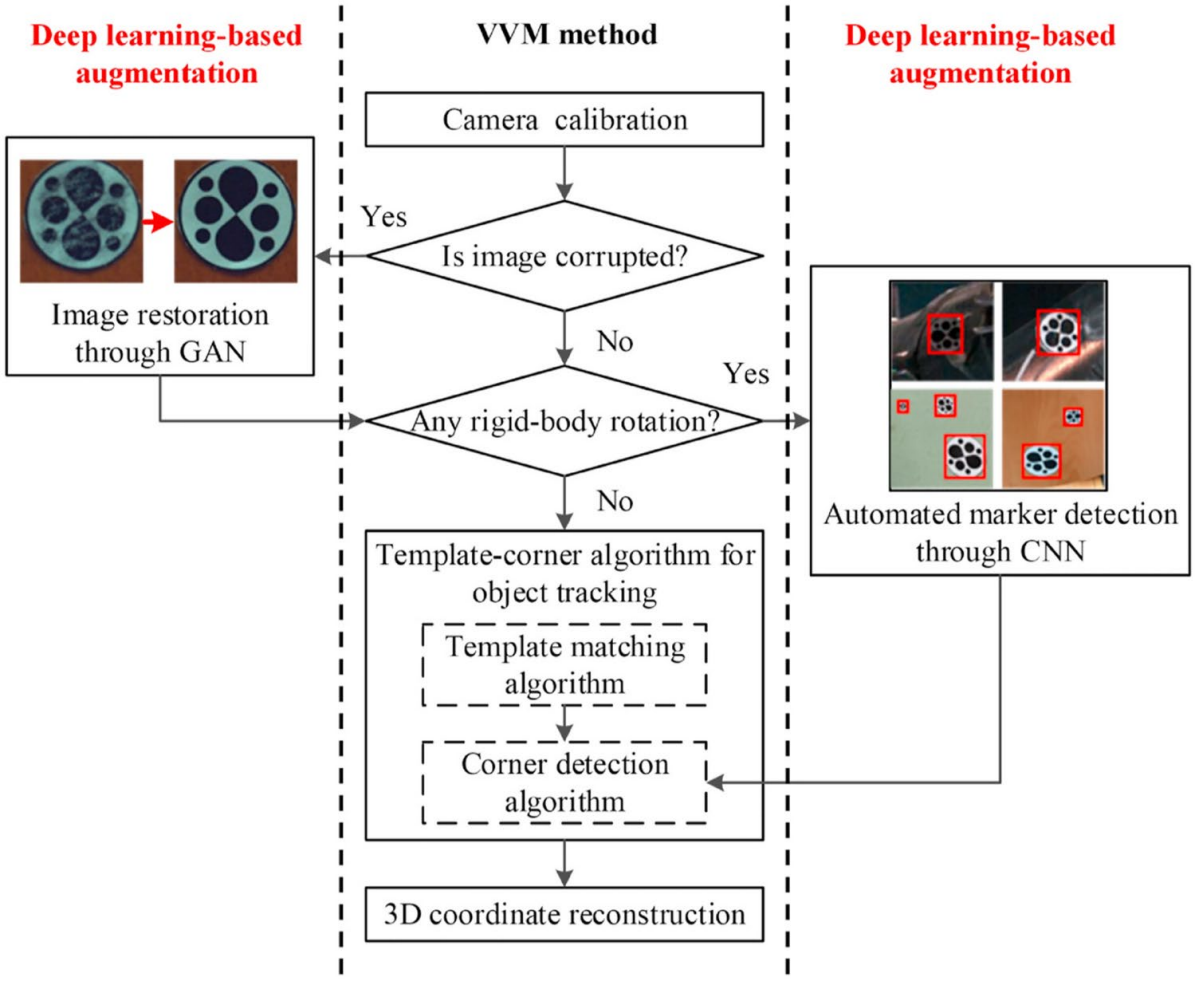
The framework of the DAVIM^45^.

**Fig. 12:**
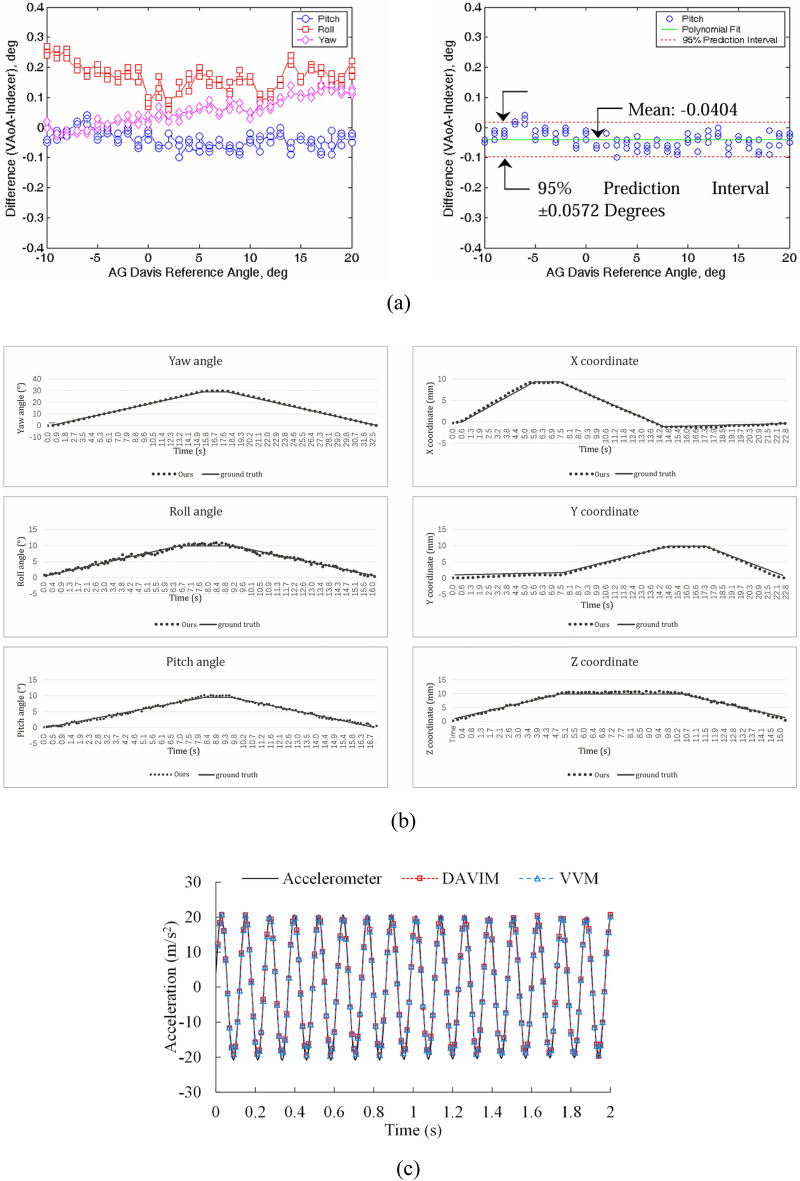
Results of different methods : (**a**) laboratory test conducted with photogrammetric and high accuracy indexer^39^ (**b**) an ultra-high precision cloud terrace with flexible control^40^ (**c**) comparison of the first 2-s acceleration time histories obtained by three different methods^45^.

In the context of wind tunnel tests conducted under harsh environmental conditions, Huang^45^ proposed a method termed Deep Learning Augmented Visual Measurement (DAVIM), as shown in Fig. 11, which integrates Convolutional Neural Networks (CNNs) and Generative Adversarial Networks (GANs). This hybrid approach enhances both the accuracy and robustness of dynamic response measurements of models. Specifically, CNNs are employed for the automated identification of designated targets, while GANs address the challenges of image corruption in severe conditions, thereby facilitating the successful identification of these targets. The DAVIM method demonstrates significantly superior performance compared to traditional sensor-based and videogrammetric measurement techniques, whose results are presented in Fig. 12c.

### Deformation measurement

Model deformation measurement is a critical aspect of the wind tunnel visual measurement system. The accuracy of dynamic tests reveals notable discrepancies: an accuracy of 0.5° is achieved when measuring the aircraft model attitude with a nominal pitch of 25°, while an accuracy of 0.18° is observed with a nominal pitch of 10°, which indicates that as the nominal amplitude increases, the measurement error also escalates^43^. Furthermore, it highlights that under wind load, the non-rigid model experiences elastic deformation. If model deformation is neglected, it can lead to significant errors in the measurement data. The deformation primarily occurs in control surfaces, such as wings and rudder surfaces, and can be quantified by analyzing the changes in spatial coordinates of multiple targets located on these surfaces. NASA has conducted numerous studies on model deformation in wind tunnels over the past 2 decades^46–49^. Similarly, the European Supersonic Wind Tunnel^50^ and the French National Center for Aerospace Research (CNERA)^51,52^ have developed vision systems for measuring model deformation. Experimental validation has demonstrated that visual measurement systems based on videogrammetric techniques hold significant promise for accurately measuring model deformation.

The fundamental principle of both deformation and attitude measurement involves using a camera to capture targets on the model. By employing the geometric projection relationship, the 2D image points can be utilized to determine the 3D coordinates of the model targets, thereby facilitating the calculation of the model’s attitude or deformation^53^. The evolution of visual systems for measuring the deformation and attitude of models in wind tunnel tests has progressed from non-contact measurements to real-time vision measurements, and ultimately to high-precision real-time measurements. The feasibility of computer vision-based measurements in wind tunnel tests is firstly validated through static experiments, while dynamic tests are conducted to estimate the methods’ performance. Higher accuracy is achieved by employing multiple cameras and more efficient image processing algorithms that enable real-time measurements.

**Fig. 13:**
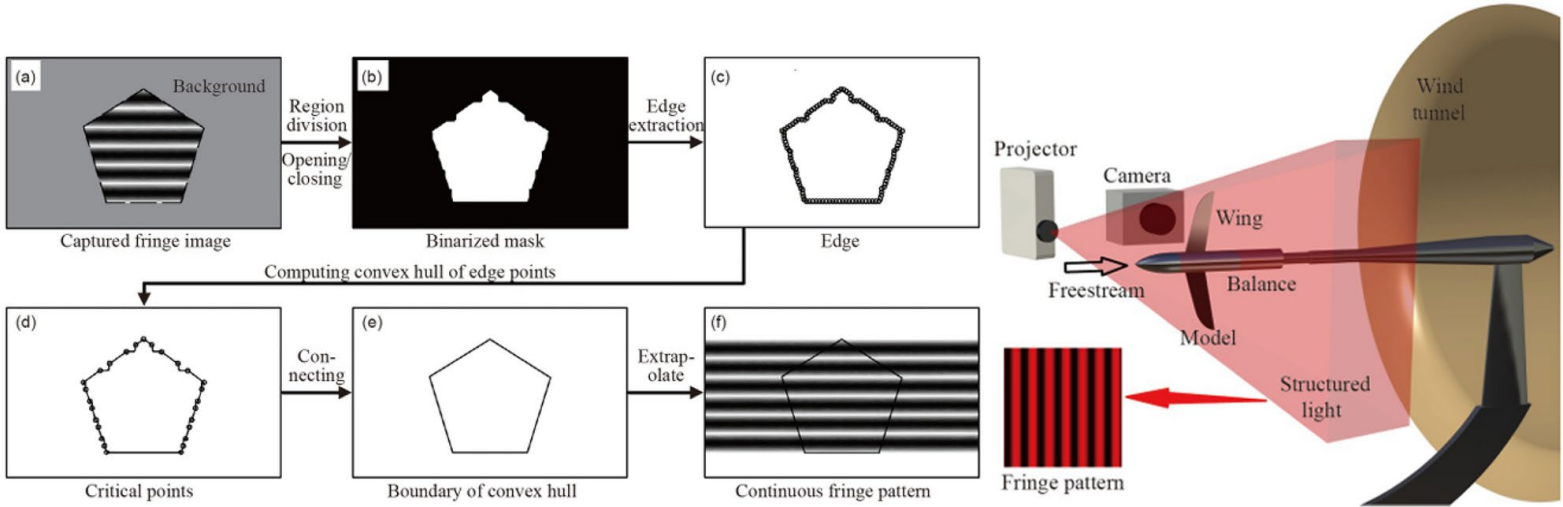
The flow diagram of the fringe pattern extrapolation and schematic sketches of the experimental setup of wind tunnel^54^.

The model’s attitude can be represented by the orientation of the rigid segments. Differing from it, the deformation of the model refers to the change in attitude of the non-rigid segments relative to the model as a whole at the actual attitude. Thus, the deformation of the non-rigid segments is characterized by the spatial changes relative to the rigid segments. Guo^54^ presents a high-precision 3D deformation measurement method known as High-Accuracy Deformation Measurement (HADM). This method leverages the principles of structured light to derive accurate phase-height mapping and fringe pattern distribution using an arbitrarily arranged projector in conjunction with monocular vision, as shown in Fig. 13. This innovative approach alleviates spatial constraints between the camera and projector, thereby enhancing practical applicability when compared to traditional Fourier Transform Profilometry (FTP). Additionally, HADM employs dynamic boundary processing algorithms to minimize deviations and uncertainties between the ideal model and real-world conditions. The average measurement uncertainty is 0.0237mm, no more than 0.01% of the side length of 25cm of the FOV.

**Fig. 14:**
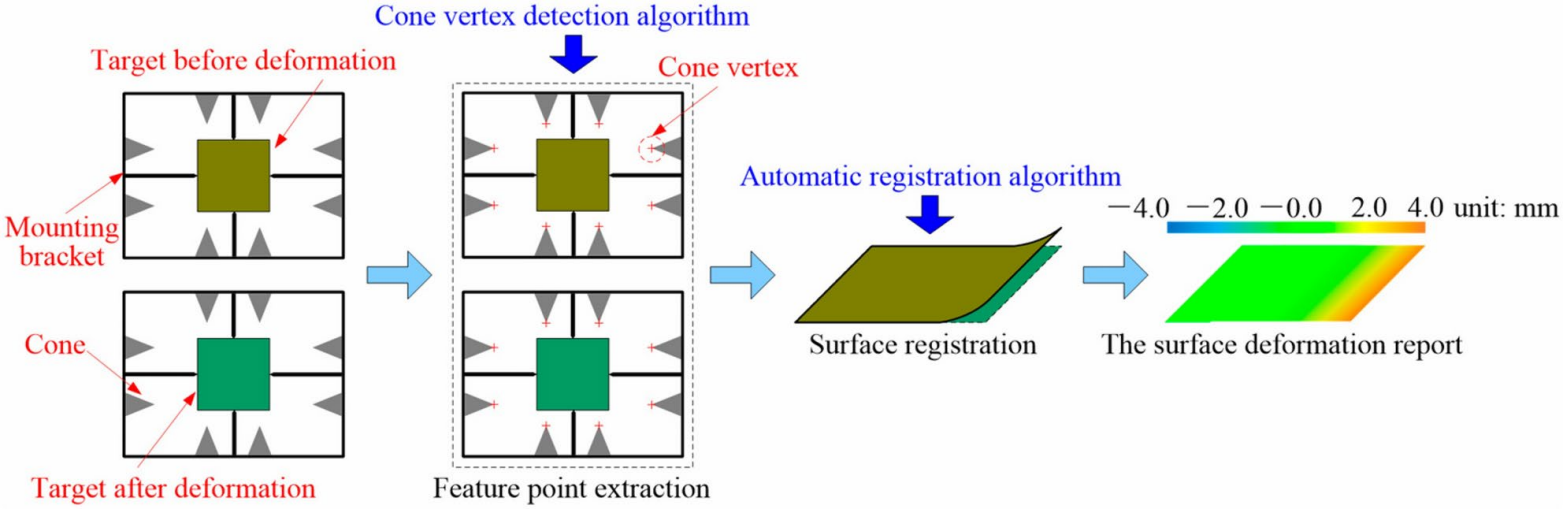
A flowchart of surface deformation measurement with cones^56^.

Yu^55^ designed a wind tunnel test deformation measurement system for a vertical propeller hovering micro air vehicle with a flexible aerodynamic shape based on stroboscopic imaging technology and binocular vision. The Scale-Invariant Feature Transform (SIFT) algorithm is used to extract image feature points for feature matching, while outliers are eliminated using the Random Sample Consensus (RANSAC) algorithm to enhance alignment accuracy. Following the calculation of the feature points’ 3D coordinates, the 3D data are fitted using the subdivision surface method to facilitate the computation and visualization of aerodynamic deformation. To address the challenge of measuring surface deformation caused by a limited overlapping area of the point cloud before and after deformation, Liu^56^ proposed an automatic point cloud alignment method inspired by biological vision. This method involved developing a camera-projector system, as shown in Fig. 14, along with securing a minimum of three cones around the target, which focused on extracting geometrical features by integrating data from multiple views. Initially, the transformation matrix between the two point clouds was computed using a random strategy to register the vertices of the cones. Subsequently, this initial alignment was refined using the Iterative Closest Point (ICP) algorithm, achieving a maximum rotational error of 0.1° and a translational error of 1 mm. To evaluate measurement accuracy, the RMS error was utilized.

## Data fusion-based measurement in wind tunnels

Different technologies exhibit distinct advantages, sensitivities to specific types of information, and inherent limitations regarding their underlying principles and conditions of use. Merely relying on a targets-based visual system to measure model attitude or deformation is insufficient, as cameras can only capture data from targets attached to the model’s surface in a certain regular distribution. This approach presents a discrete characteristic that fails to capture the deformation of non-rigid surfaces between the targets. With the advancement of computer vision-based measurement technology, it has become essential to employ multiple techniques concurrently during wind tunnel tests. The fusion of various independent techniques enhances the comprehensiveness of the measurement system.

Data fusion involves acquiring information through different technologies and combining the characteristics across multiple dimensions. This approach capitalizes on the complementary advantages of diverse information sources, effectively improving the accuracy and robustness of the measurement system and enhancing its overall stability.

### Wind tunnel flow diagnostics and visualization technology

**Fig. 15:**
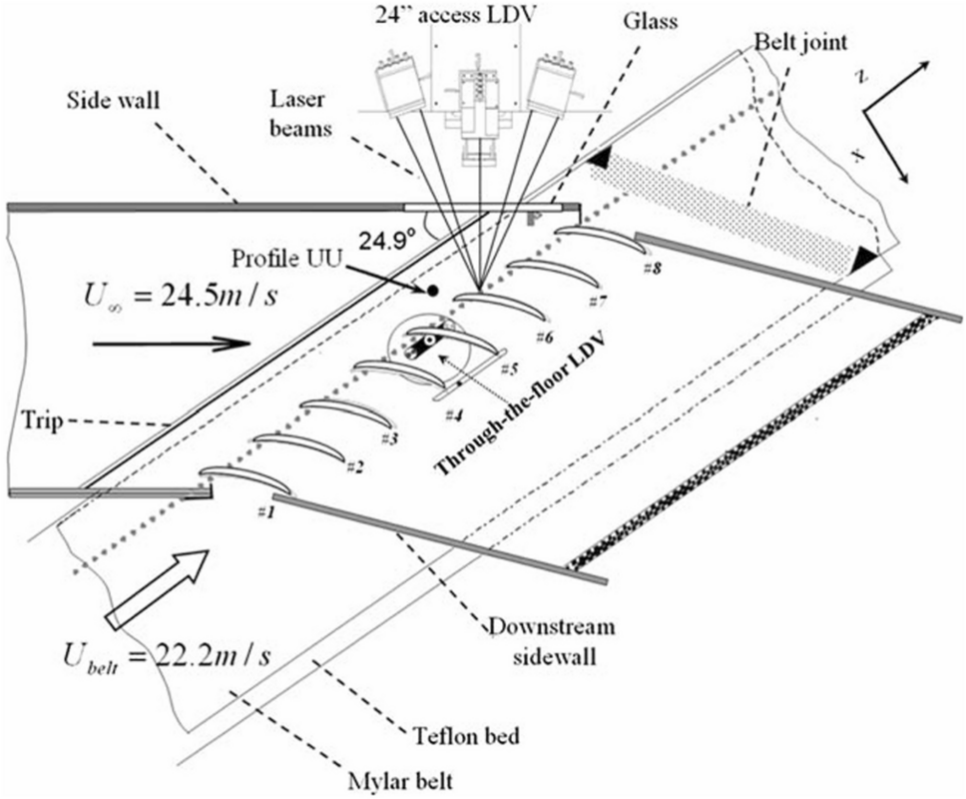
LDV experimental equipment in wind tunnels^58^.

The purpose of flow visualization is to visualize fluid movement phenomena. Traditional flow visualization methods don’t incorporate measurement capabilities, they solely illustrate the airflow characteristics within wind tunnels through intuitive physical visualizations, such as the smoke flow method, helium bubble method, and oil flow method. The advent of computer vision has augmented traditional flow visualization techniques, enabling the acquisition of richer flow field information. Through the application of image processing, various colors can be utilized to represent parameter variations, facilitating the depiction of the flow field in wind tunnel images displayed on computers. This improvement enhances both the speed of flow visualization processing and the quality of the images produced. Schlieren graph is one of the most commonly used flow visualization methods in wind tunnel tests, the principle of which is to transform the light disturbed by the flow field into the light intensity distribution recorded on the image plane. As flow visualization technology continues to advance, a range of flow diagnostics and visualization technology has emerged, such as laser-induced fluorescence (LIF)^57^, laser-Doppler velocimeter (LDV)^58^, particle image velocimetry (PIV)^59^, pressure-sensitive paint (PSP)^60^ and temperature-sensitive paint (TSP)^61^ measurements. These methodologies possess the capability for both qualitative visualization and quantitative measurement, catering to the demands of assessments in the complex airflow environments typical of wind tunnels. Schematic diagrams of LDV are illustrated in Fig. 15.

### Fusion with flow diagnostics and visualization technology

In wind tunnel visual measurement tasks, the fusion of existing flow diagnostics and computer vision-based measurement enables the simultaneous measurement of flow characteristics and model attitude or deformation, which enhances the efficiency of wind tunnel testing. Furthermore, the ability to cross-verify different measurement data ensures the accuracy of the results. James^62^ proposed a data fusion method for wind tunnel testing that combines videogrammetry measurement techniques with PSP to synchronously capture surface pressure and model deformation. This approach utilizes a single camera and light source for both methods, thereby simplifying experimental equipment. Additionally, by analyzing the pressure field data obtained from the PSP and incorporating the material properties of the model, it is possible to calculate the deformation field of the model under wind load, the data fusion of two methods enhances the accuracy of deformation measurements^35,63^. However, simultaneous measurements have not yet been achieved in this research, indicating potential for further development. Burner^64^ proposed a visual measurement system combining TSP and videogrammetry for simultaneous measurement of model deformation and flow transition. In this case, the TSP is used for transition detection in the boundary layer, and the videogrammetry measures the deformation and torsion of the model’s main wing and flaps. The deformation measurement system is adapted to UV illumination, which ensures that high-contrast images can be obtained during TSP data acquisition, and by setting the parameters of the measurement system, it is possible to realize the use of the normal test section illumination when TSP data are not being acquired.

In the realm of data fusion, notable incompatibilities persist between two prevalent techniques: PSP/TSP and videogrammetry. PSP/TSP necessitates specific wavelength illumination with distinct spectral characteristics and high-resolution cameras^65,66^. Conversely, videogrammetry requires the acquisition of high-contrast target images and high-speed cameras, which are largely insensitive to spectral characteristics. Consequently, it is imperative to optimize hardware equipment to enable both techniques to share a unified vision system, thereby facilitating synergistic interaction and mutual non-interference. This fusion aims to achieve real-time, simultaneous, and high-precision measurement of characteristics related to pressure or temperature and deformation^67^. However, it is important to note that PSP/TSP is susceptible to being washed away by airflow during wind tunnel tests conducted at high Mach numbers^68^, which limits the feasibility of fusing PSP/TSP and videogrammetry methods in high-speed wind tunnel environments.

**Fig. 16:**
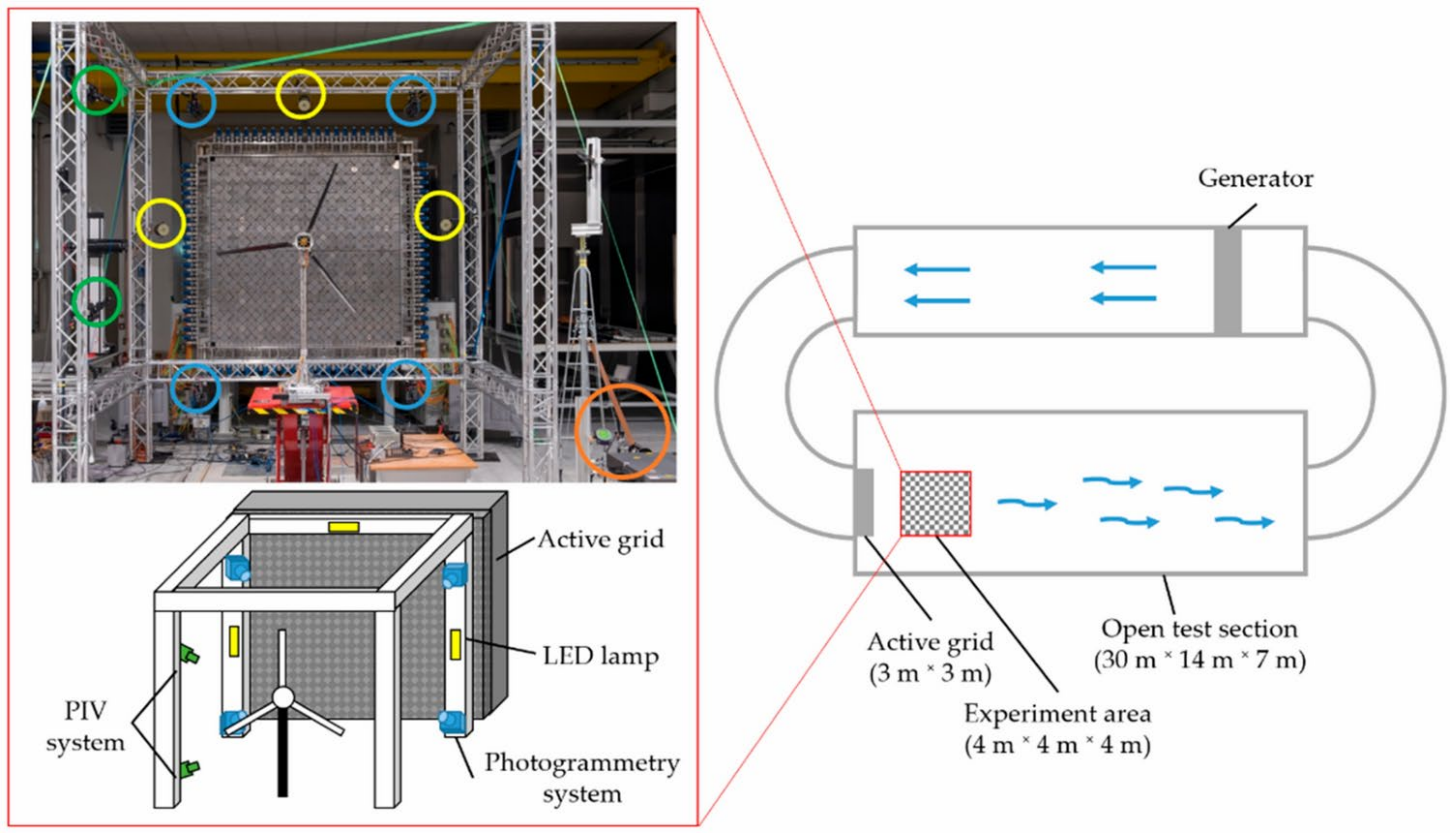
Experimental setup in the wind tunnel and Top view of the wind tunnel. PIV cameras are encircled in green, PIV laser in orange, photogrammetry in blue, and LED lamps in yellow^69^.

Nietiedt^69^ proposed a system that integrates PIV with trinocular vision for wind tunnels characterized by coupled fluid–solid interactions, which can achieve simultaneous measurement of fluid velocity and model deformation through the concurrent fusion of temporal and spatial data, as shown in Fig. 16. Cheng^70^ developed a model attitude measurement method utilizing the Schlieren graph and monocular vision. Firstly, the contour of the model is extracted from both the Schlieren graph and the images captured by the camera to calculate the initial value of the model’s attitude. The initial value is then matched with the Computer-Aided Design (CAD) model to determine the accurate attitude. In particular, this method requires only the orientations of the camera and the Schlieren graph instrument, eliminating the need for fixed installation in specific positions. This enhances the flexibility of the system, achieving an accuracy of up to 0.1°. Dong^71^ developed a novel speckle-patterned PSP system that integrates the single-shot lifetime method, binocular vision, and digital image correlation (DIC) technology to realize the simultaneous measurement of pressure and deformation fields of high-speed rotating blades in wind tunnels. The single-shot lifetime method applies high-energy laser pulses to excite the PSP, capturing images from different angles by randomly printing a speckle pattern on the surface of the PSP, which facilitates pixel-level image alignment for the reconstruction of the pressure field. Ultimately, point cloud data are generated through cross-correlation analysis and triangulation, which are then fused with CAD model data to obtain deformation and pressure data. The measurement deviation of the deformation field and the average standard deviation (STD) were 0.13 mm and 200 Pa respectively.

### Fusion with CAD model

**Fig. 17:**
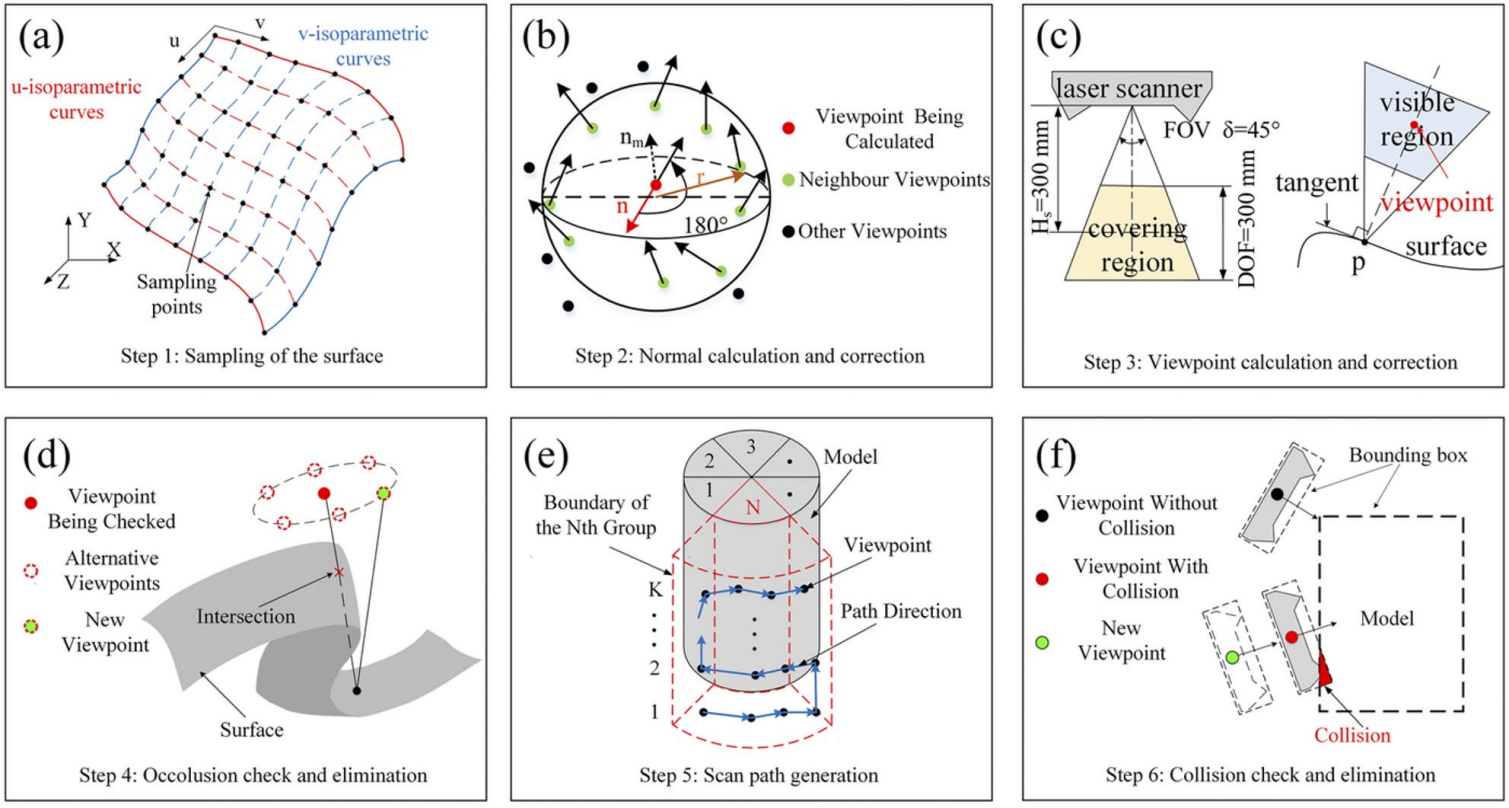
Six-step procedure of scan path generation^44^.

With advancements in fusion technology, CAD is increasingly integrated into wind tunnel visual measurement systems. CAD provides a virtually error-free model, thus, extracting data from CAD models allows for more accurate characterization of the physical models, thereby enhancing the precision of wind tunnel visual measurement. Zhang^44^ developed a trinocular visual measurement system that adopts a CAD model transformed into a non-uniform rational B-spline (NURBS) surface to measure the position and attitude of the model. As shown in Fig. 17, this system collects a set of points from the NURBS surface, which are then used as viewpoints for the laser scanner, enabling the automatic generation of scanning paths to efficiently establish the model coordinate system. Kasula^72^ designed a target-free model attitude measurement method based on binocular vision combined with CAD, exploiting Harris corner points to detect key feature points in the image while tracking these corners in subsequent video sequences by the Kanade–Lucas–Tomasi algorithm. Finally, the attitude angle of the model is calculated using quaternions. What the extended Kalman filter does is to integrate the inertial measurement unit on the model, potentiometer gimbal, image feature data, and CAD model to improve the robustness and accuracy of the system, and the dynamic root mean square errors of roll rate, pitch rate and yaw rate reached 0.0090 rad/s, 0.0262 rad/s, and 0.0034 rad/s, respectively. Comparision of Data fusion-based methods used in test of wind tunnel is shown in Table 2.

**Table 2:** Comparision of Data fusion-based methods used in test of wind tunnel.

	Author	Principles of vision	Methods (videogrammetry fuses with)	Performance
Fusion with flow diagnostics and visualization	James^62^	Monocular vision	PSP	Synchronous measurement of pressure and deformation, but not simultaneous
	Burner^64^	Binocular vision	TSP	Simultaneous or individual measurement of deformation and flow transition
	Nietiedt^69^	Trinocular vision	PIV	Simultaneous measurement of fluid velocity and deformation
Both	Cheng^70^	Trinocular vision	Schlieren graph + CAD	Measurement of the model’s attitude with an accuracy of 0.1°
	Dong^71^	Binocular vision	PSP + DIC + CAD	Synchronous measurement of pressure and deformation with an accuracy of 200Pa and 0.13mm
Fusion with CAD	Zhang^44^	Trinocular vision	DAVANet + NURBS surfaces + CAD	Measurement of the model’s attitude
	Kasula^72^	Binocular vision	KLT + Kalman filter + CAD	Measurement of the attitude with dynamic RMS errors of roll, pitch and yaw rate reached 0.0090 rad/s, 0.0262 rad/s, and 0.0034 rad/s

## Challenge

The application of videogrammetry techniques for measuring model deformation and assessing uncertainty in the attitude angles of wind tunnel models is thoroughly examined under both static and dynamic conditions^73–75^. The analysis includes a sensitivity assessment focused on deriving 3D spatial coordinates from 2D image coordinates, which is crucial for ensuring measurement fidelity, and highlights the dynamic effects on model deformation and the accuracy of attitude. Through this research, a clearer understanding of these challenges is achieved, providing valuable insights for advancing vision system development within wind tunnel tests. Contrasting with conventional visual measurement environments, wind tunnels present unique challenges due to their constrained and turbulent airflow conditions. The interaction between the airflow and the test model generates intricate aerodynamic phenomena that can significantly impact measurement accuracy. These complexities complicate visual measurements and necessitate innovative approaches to improve the reliability and precision of the computer vision-based measurement employed in such environments. With the advancement of wind tunnel visual measurement systems, research has shifted from solely focusing on technology to including error analysis of wind tunnel problems. Challenges such as model vibration and observation window distortions have been identified, leading to targeted solutions that enhance measurement accuracy. These improvements not only refine the measurement precision but also optimize the overall wind tunnel visual measurement system.

### Impact of vibration

There are two types of vibration in wind tunnel tests, one is the vibration of the test section and the other is the vibration of the support mechanism and model^44,76^. The optical pathway within the wind tunnel is constrained, necessitating that the camera captures images of the model through an observation window. Aimed to maximize the utilization of camera resolution, the camera is positioned close to this window. Consequently, vibrations from the test section can induce camera vibrations, resulting in changes to its position and orientation alterations that affect the external parameters and introduce significant errors in the measurement of three-dimensional spatial coordinates. The second type of vibration arises from the aerodynamic load associated with the incoming airflow in the wind tunnel test section, which leads to vibrations in the model, thereby impacting measurement accuracy.

**Fig. 18:**
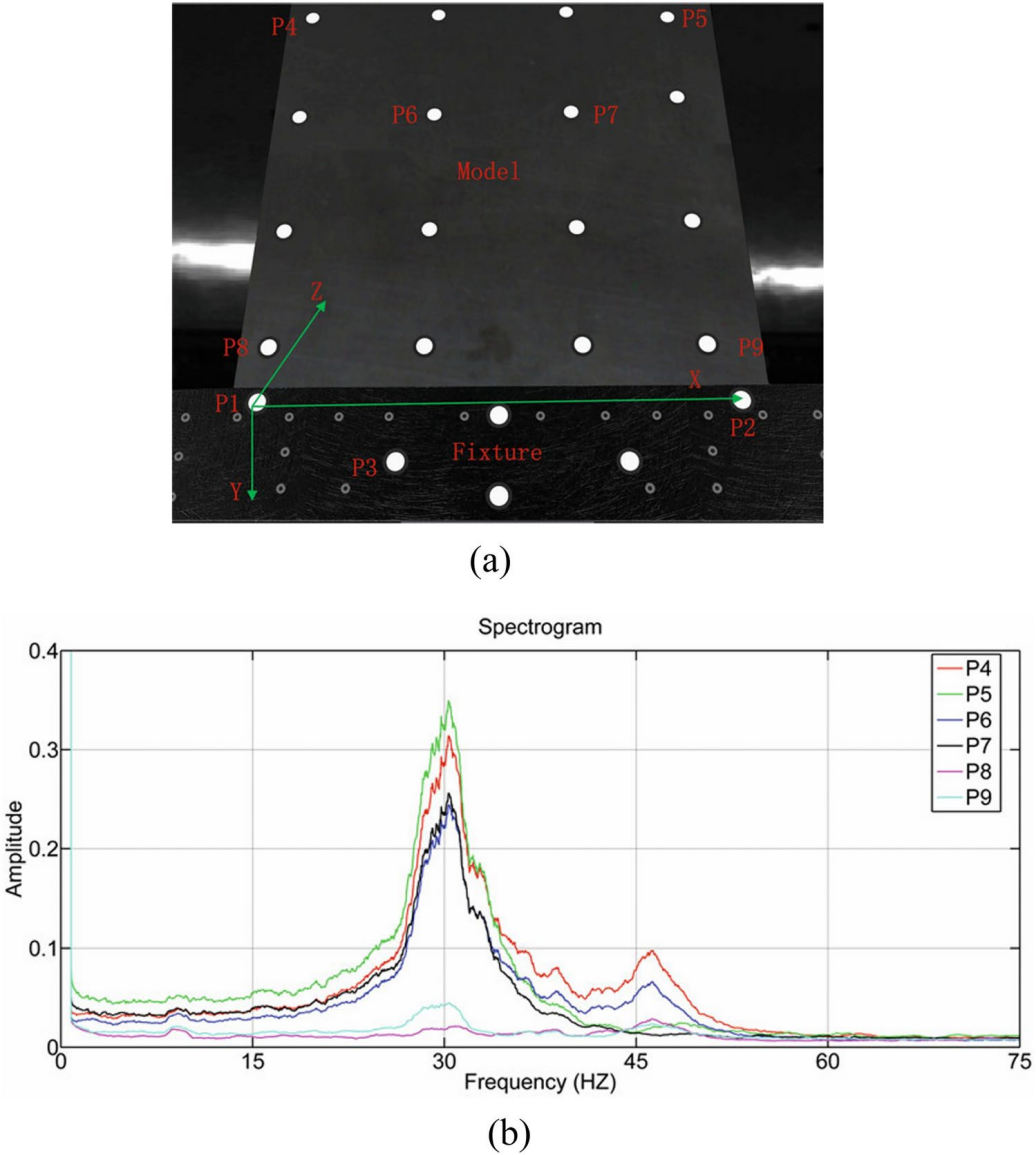
Chen’s method: (**a**) the measuring model, (**b**) the spectrogram of P4, P5, P6, P7, P8, and P9^81^.

Traditional camera calibration typically involves the use of calibration targets with known three-dimensional structures, such as calibration blocks or checkboards. However, these methods are inadequate for addressing the challenges posed by the first vibrations during the measurement process in a vision system. A self-calibration approach can be employed to mitigate the effects of the first vibration-induced changes in the camera’s external parameters^77–80^, which doesn’t require auxiliary calibration targets and can be executed concurrently with the camera’s visual tasks compared to conventional calibration methods. This characteristic makes it particularly suitable for continuously operating visual systems. Notably, self-calibration can achieve near real-time processing speeds, allowing for the adjustment of the camera’s external parameters in response to vibrations, thereby ensuring measurement accuracy. Chen^81^ proposed an innovative two-step calibration method leveraging camera self-calibration techniques to mitigate the impact of camera vibrations on measurement accuracy. The methodology commences with a rapid, non-iterative process to derive the initial estimates of the camera’s orientation. This is followed by the application of a globally convergent orthogonal iterative approach to refine these estimates, resulting in an accurate final solution. Operating at a camera frame rate of 150 Hz, the method was validated through wind tunnel experiments, where it demonstrated a significant enhancement in measurement precision for the vision system at a wind speed of Mach 5. The findings revealed a substantial reduction in the maximum measurement error, decreasing from 0.516 to 0.044 mm. Furthermore, the calibrated system exhibited improved accuracy and stability, particularly in the presence of high levels of noise. Additionally, the model’s vibration frequency was quantitatively analyzed as shown in Fig. 18.

The interaction between an aircraft’s structural characteristics and aerodynamic nonlinear forces induces vibrations in the model, which leads to discrepancies between the measured data and theoretical predictions, thereby introducing errors into the test outcomes. In order to address these challenges, it is essential to conduct vibration analysis concurrently with the measurement of the model data. By integrating vibration analysis, researchers can better calibrate the system data, enhancing the accuracy and reliability of the measurements, which not only improves the fidelity of the experimental data but also facilitates the validation and optimization of the model design^82–84^.

**Fig. 19:**
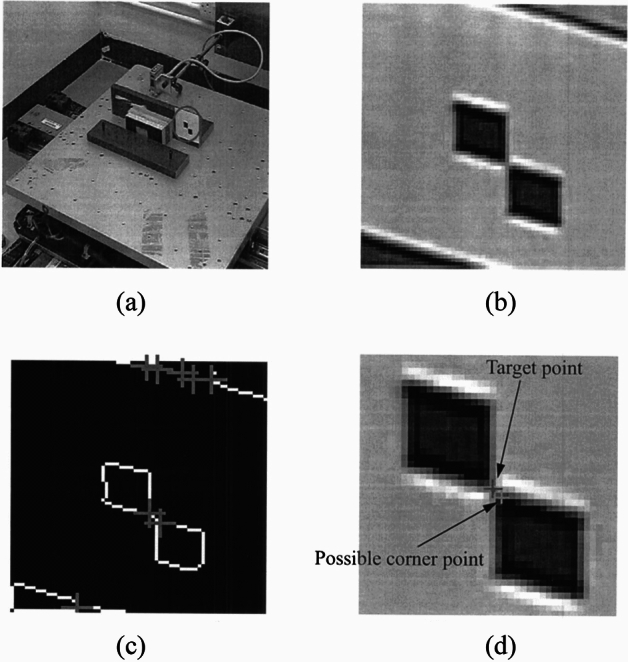
Target point extraction: (**a**) target pattern, (**b**) zoom-in of target pattern, (**c**) intersection of image skeleton, (**d**) detected target point^86^.

**Fig. 20:**
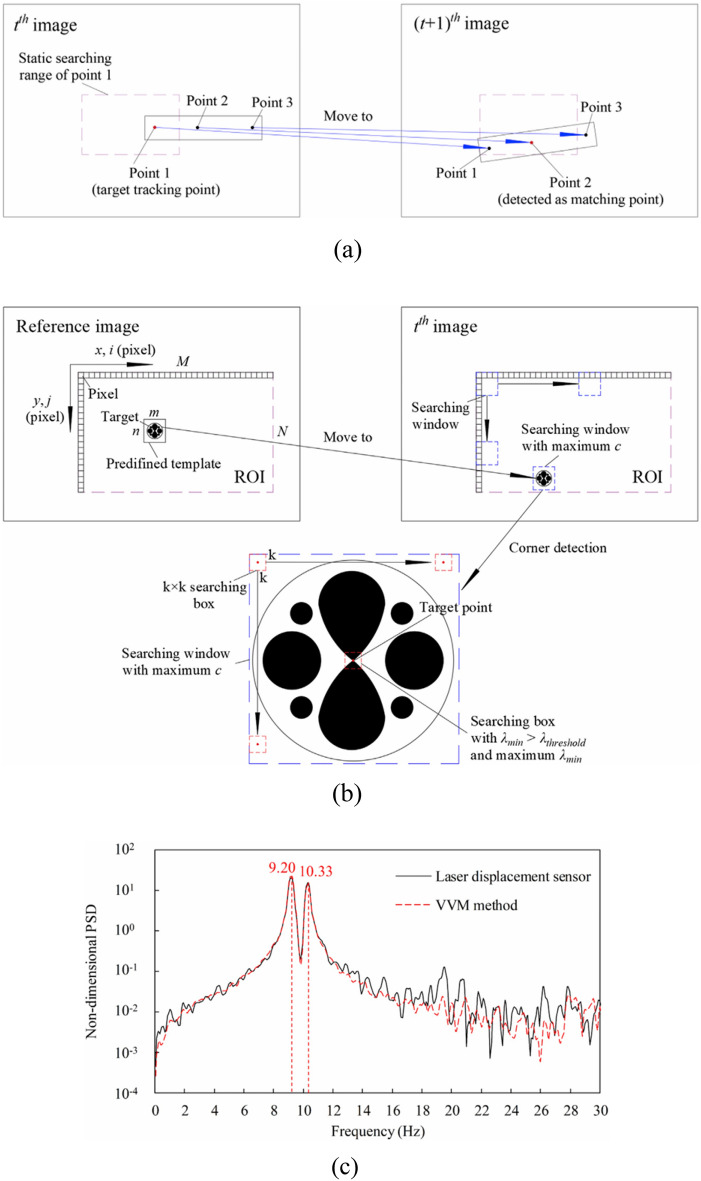
(**a**) An example of mismatching problem of the corner detection algorithm (**b**) process of the template-corner algorithm (**c**) comparison of Non-dimensional PSD functions obtained by the laser displacement sensor and VVM method^87^.

**Fig. 21:**
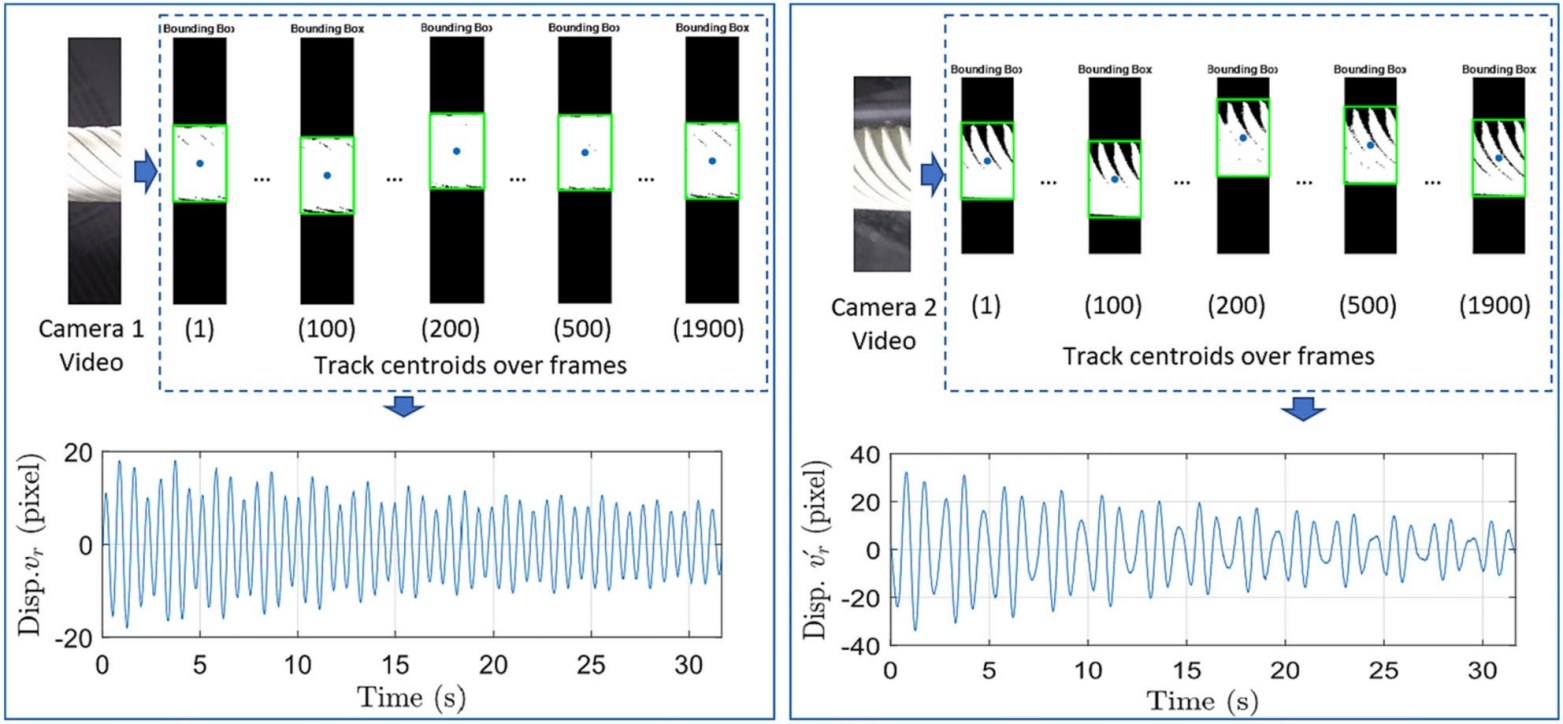
Pixel displacements from two camera videos^88^.

Zhu^85^ measured the vibration of a lightweight flexible wing in a wind tunnel test by using the videogrammetric measurement method and analyzed the dynamic characteristics of the wing such as the vibration frequency and the amplitude, in which the frequency measurement relative precision is superior to 0.5%, its absolute precision is superior to 0.1 Hz in a low-frequency range. Chang^86^ carried out a static single-point sinusoidal function test and wind tunnel dynamic test for the vibration of the model in the wind tunnel test based on computer vision, identified and located the target by Harris corner point detection technique as shown in Fig. 19, finally tracked the motion of the target point using the correlation method. The static experiments showed that the measurement error was small at 0.5 Hz low frequency, with an average value of 0.12–0.29mm and a standard deviation of 0.4 mm. However, it was observed that the standard deviation increased to 1.4 mm at a high-frequency vibration of 5 Hz, indicating that measurement error tends to escalate under high-frequency vibration conditions. Despite this increase in error, the dynamic vibration experimental data collected from the wind tunnel exhibited a strong consistency with the results obtained from the sensor measurements. This agreement suggests that the measurement system maintains acceptable accuracy levels under these challenging conditions, effectively meeting the established requirements for reliability and precision in the data collected during the experiments. Huang^87^ proposes a vibration measurement method based on the template corner-point algorithm, which combines the advantages of the template matching algorithm and the corner-point detection algorithm for accurately tracking the target point as shown in Fig. 20. The measurement results are consistent with the displacement and acceleration sensors. Le^88^ employs a novel approach that integrates computer vision with phase-based video motion magnification (PVMM) to effectively capture the vibrations of a model within a wind tunnel environment. This method utilizes the center-of-mass-based bounding box tracking (CBBT) technique with two cameras to accurately track the model’s motion across each video frame as shown in Fig. 21. From the magnified video, the model’s time-range displacements are extracted, allowing for a detailed analysis of its vibrational behavior. Subsequently, the intrinsic frequency and damping ratio of the model are derived from the displacement data using the Wavelet Screening Random Decrement Technique (WS-RDT). The results demonstrate high precision, with measurement errors of only 0.003 Hz for the intrinsic frequency and 0.19 for the damping ratio, which not only enhances the understanding of the model’s vibrational characteristics but also provides a robust framework for analyzing dynamic behavior in complex aerodynamic environments.

### Distortion of the observation window

In the wind tunnel test section, the observation window serves as the sole access for capturing images of the model. Typically, the thickness of this window exceeds that of conventional glass to withstand the strong wind loads present in testing environments. However, this increased thickness can lead to distortions of the observation window during the imaging process^89,90^. These distortions can significantly impact the accuracy of measurements obtained from the vision system, introducing errors that may compromise the reliability of the data collected during tests.

**Fig. 22:**
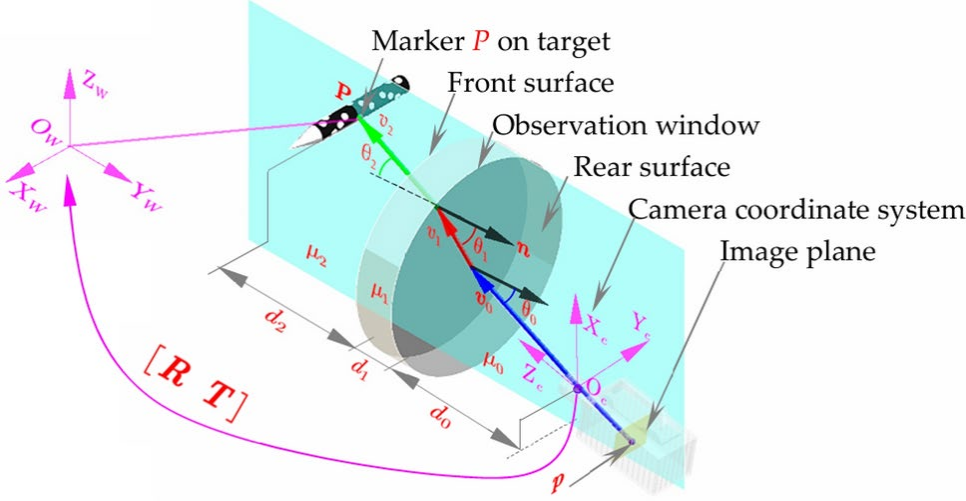
Principle of the attitude measurement model^93^.

Ma^91^ introduced a multi-layer refraction photogrammetry model utilizing binocular vision with multi-layer refractive geometry to effectively account for light refraction caused by the observation window between the model and the camera. The approach employs Zhang’s calibration method to initially calibrate the camera, which then calibrates the thickness of the observation window and the direction vectors of each segment of the optical path^92^. To enhance the accuracy of this calibration, the system optimizes these parameters by leveraging constraints derived from the distances between points on a calibration target. This innovative calibration process enables high-precision measurement of the multi-layer photographic model. Experimental validation of this method reveals a maximum displacement error of just 0.11 mm. Additionally, the method achieves notable accuracy in attitude measurements, with maximum deviations of 0.1° for pitch angle, 0.11° for yaw angle, and 0.32° for roll angle. Liu^93^ employed computer vision to quantify the model attitude, taking into account the refraction of the observation window as shown in Figure 22. Furthermore, an imaging model was proposed that incorporates the effect of refracted light. Initially, the internal matrix of the camera was calculated, followed by the determination of the normal vectors of the observation window to derive the external parameters and the thickness of the dielectric layer. Eventually, a matching method based on coplanar constraints was used to achieve the attitude estimation. The experimental results demonstrated that the displacement and angular accuracies were enhanced by 57% and 33.6%, respectively. The comparision of approaches adopted for vibration and the observation window in test of wind tunnel is shown in Table 3.

**Table 3:** Comparision of approaches adopted for vibration and the observation window in test of wind tunnel.

	Author	Methods	Performance
Vibration	Chen^81^	Self-calibration	the maximum measurement error decreases from 0.516 mm to 0.044 mm
	Zhu^85^	Videogrammetry	frequency measurement relative precision is superior to 0.5%, absolute precision is superior to 0.1Hz
	Chang^86^	Harris corner point detection	measurement error was 0.5Hz with an average value of 0.12mm to 0.29mm and a standard deviation of 0.4mm
	Huang^87^	Template corner-point	results are consistent with the displacement and acceleration sensors
	Le^88^	PVMM + CBBT + WS-RDT	measurement errors of only 0.003 Hz for the intrinsic frequency and 0.19 for the damping ratio
Distortion of the observation window	Ma^91^	Multi-layer refraction photogrammetry model	maximum displacement error of just 0.11 mm. maximum deviations of 0.1° for pitch angle, 0.11° for yaw angle, and 0.32° for roll angle
	Liu^93^	Nonlinear camera calibration model	displacement and angular accuracies were enhanced by 57% and 33.6%, respectively

## Perspectives

Currently, the specificity and complexity of engineering applications present significant challenges, with many studies remaining at the simulation stage and lacking reliable experimental validation. Therefore, a comprehensive analysis of the current state of the art reveals several future development trends for wind tunnel attitude and deformation measurement technologies based on computer vision:

1. *Adaptation to specialized application scenarios* There is a need for computer vision-based measurement methods to be tailored to the unique conditions of high-speed environments, restricted visual access, and limited space inherent in wind tunnels. Additionally, the vibrations induced by wind loads on both the model and the camera, along with distortions of the observation window, impose considerable deviations on visual information processing. At the same time, the lightening of the measured object and the mitigation of reflections from the metallic surfaces present a significant challenge. Consequently, it is essential to take these specific characteristics and constraints into account when developing measurement technologies for model attitude and deformation.
2. *Reliability and precision in real-time measurements* The ability of computer vision-based measurements to meet accuracy requirements is pivotal for achieving contactless measurement. Utilizing high-resolution imagery can enhance measurement precision across both close-range and wide-field applications, however, this often leads to challenges in real-time performance due to increased computational demands. Therefore, lightweight vision algorithms are crucial for balancing high accuracy with real-time processing capabilities.
3. *Fusion of multimodal information* Relying solely on a single source of information for model attitude and deformation measurements presents limitations. The discrete nature of visual measurement methods, particularly those based on targets, can introduce errors in calculating the model’s center point and fitting the deformation between the target points. Thus, effectively leveraging the strengths of various technologies can compensate for the deficiencies in multidimensional information. At the same time, the wind tunnel test preparation process is complex, synchronization of different technologies can greatly improve the efficiency of the wind tunnel test.
4. *Combining with deep learning methods* There is little application of deep learning methods for measuring model attitude and deformation in wind tunnel tests. Future research on deep learning-based visual measurement systems for wind tunnels should be further developed, such as image processing^94–97^, monocular depth^98–100^, and pose estimation^101–103^, to achieve high-precision and real-time intelligent measurements. At the same time, transfer learning^104–106^ develops target networks for different model types with similar features in varying test environments, which can help overcome the limitations associated with the demand for large data samples, thereby enhancing measurement efficiency and performance.
5. *Tending to be efficient and comprehensive* It takes a long time to prepare a wind tunnel test. At present, the mainstream wind tunnel visual measurement methods are based on targets, and the time consumed by attaching targets will affect the efficiency of tests. In addition, when models are very small, it is required that the targets must be small enough, which will affect the measurement accuracy and also lead to the loss of targets. Therefore, it is necessary to research some methods that do not rely on targets to cope with various measurement situations in wind tunnel tests.

## Conclusion

Computer vision-based models for attitude and deformation measurements play a crucial role in wind tunnel testing, offering advantages such as non-contact operation, automation, visualization, flexible configuration, and high accuracy in near-field measurements. Advancements in the technology are addressing the limitations of traditional sensor measurements, significantly enhancing the accuracy of aerodynamic parameter assessments for aircraft, which offers more precise and reliable data for aircraft design. Additionally, the configuration of multi-source vision measurements can be adapted for various environments, ranging from subsonic to supersonic conditions, while improving the robustness of the system. This adaptability not only shortens the preparation time for wind tunnel tests but also improves overall testing efficiency.

This paper systematically reviews the research advancements in attitude and deformation measurement methods within wind tunnels and summarizes representative methodologies and findings. This paper first provides a brief history of the wind tunnel vision measurement system, followed by a discussion of its components. Subsequently, a theoretical model of the wind tunnel vision measurement system is established, introducing the fundamental principles involved. Different methods for measuring model attitude and deformation are then categorized based on the distinct characteristics of the wind tunnel vision measurement system. These include non-real-time wind tunnel vision measurement methods, real-time wind tunnel vision measurement methods, and those based on deep learning. Then, these methods evolve from single-source information to multi-source information, forming data fusion, which included fusion with flow diagnosis techniques and CAD models. Finally, we identify the challenges faced by the vision measurement system during wind tunnel tests, specifically addressing issues related to camera and model vibrations, as well as distortion of the observation window.

## Data Availability

The datasets used and/or analysed during the current study available from the corresponding author on reasonable request.
